# Characterization of Giant Myoviridae With Therapeutic Potential Against Grouper Pathogen, *Vibrio alginolyticus*

**DOI:** 10.1155/ijm/3932069

**Published:** 2026-05-05

**Authors:** Mohammad Tamrin Mohamad Lal, Rafidah Othman, Elliecpearl Jasca Joning, Gilbert Ringgit, Nor Azman Kasan, Motohiko Sano, Ibnu Bangkit Bioshina Suryadi, Attabik Mukhamad Amrillah, Julian Ransangan

**Affiliations:** ^1^ Borneo Marine Research Institute, University of Malaysia Sabah, Kota Kinabalu, Sabah, Malaysia; ^2^ Biotechnology Research Institute, University of Malaysia Sabah, Kota Kinabalu, Sabah, Malaysia, bri.nrc.ca; ^3^ Institute of Tropical Aquaculture and Fisheries, University of Malaysia Terengganu, Kuala Terengganu, Terengganu, Malaysia, umt.edu.my; ^4^ Laboratory of Fish Pathology, Department of Marine Biosciences, Tokyo University of Marine Science and Technology, Minato, Tokyo, Japan, kaiyodai.ac.jp; ^5^ Department of Fisheries, Faculty of Fisheries and Marine Science, Padjadjaran University, Bandung, West Java, Indonesia, unpad.ac.id; ^6^ Department of Aquatic Resources Management, Faculty of Fisheries and Marine Science, Brawijaya University, Malang, East Java, Indonesia, ub.ac.id

**Keywords:** bacteriophage therapy, host specificity test, one step growth, T4-like virus, transmission electron microscope

## Abstract

**Background:**

*Vibrio alginolyticus* is a gram‐negative bacterium responsible for mass mortality in cultured groupers, leading to significant economic losses in the aquaculture industry. Although preventive measures such as chemical treatments, antibiotics, and pesticides have been employed, these methods have been reported to be toxic to the environment and contribute to the development of antimicrobial resistance (AMR) in treated fish.

**Methods:**

In this study, a giant Myoviridae phage strain (ValKK1‐20) was isolated from the sandy area of Kota Kinabalu, Sabah, Malaysia. The purified phage was characterized based on transmission electron microscopy (TEM) and its genomic DNA. The adsorption assay and one‐step growth analyses were conducted to predict the critical phase of ValKK3 infection.

**Results:**

TEM analysis revealed that the phage possessed an elongated head with a sheathed tail, resembling members of the T4‐like Myoviridae group. The genome characterization revealed that the phage belongs to T4 phage with a genome size 248,088 bp with 41.2% *G* + *C* content and 390 predicted open reading frames. Additionally, it demonstrated a short eclipse period of 36 min and a latent period of 48 min, with a large burst size of approximately 174 plaque‐forming units (PFU) per cell.

**Conclusions:**

In conclusion, this study highlights the potential of selected phage ValKK3 as a biocontrol agent against *V. alginolyticus*, offering a promising alternative for sustainable aquaculture management.

## 1. Introduction

*Vibrio* bacteria are widely distributed in marine and estuarine environments. Pathogenic *Vibrio species* have been identified, including *Vibrio alginolyticus* [1], *V. parahaemolyticus* [2], *V. harveyi* [3], *V. vulnificus* [4], *V. carchariae* [5], and *V. alginolyticus* [6]. Among these species, *V. alginolyticus* is known to infect marine organisms due to its high species diversity [7, 8], leading to rapid fish mortality [9] and significant economic losses in aquaculture [10, 11]. This pathogen has been reported to cause mass mortality in cultured gilthead seabream (*Sparus aurata*) [12], sharpsnout seabream (*Diplodus puntazzo*) [13], seahorse (*Hippocampus reidi*) [14], and Asian seabass (*Lates calcarifer*) [15]. Furthermore, *V. alginolyticus* is associated with various *Vibrio*‐related diseases affecting fish, crustaceans, and mollusks [16–19].

Global protein consumption is projected to increase by 59% by 2030, with grouper fish being one of the most economically valuable species since the 1970s, particularly in Singapore, Malaysia, Hong Kong, Thailand, and Taiwan [20]. *V. alginolyticus* poses a significant threat to grouper survival due to its toxic extracellular products. Recent studies have highlighted the economic losses in grouper aquaculture caused by vibriosis outbreaks [10]. *V. alginolyticus* outbreaks are responsible for high mortality rates in *Epinephelus* species, including brown‐marbled grouper (*Epinephelus fuscoguttatus*) [21], orange‐spotted grouper (*Epinephelus coioides*) [22], longtooth grouper (*Epinephelus bruneus*) [23], and Malabar grouper (*Epinephelus malabaricus*) [24]. Major outbreaks of *V. alginolyticus* have been reported in Malaysia and China. In Malaysia, outbreaks in grouper aquaculture account for 19% of infections, with mortality rates reaching up to 50% in deep‐sea cages in Langkawi [25]. This finding aligns with studies on hybrid grouper (♀*E*.*f* *u* *s* *c* *o* *g* *u* *t* *t* *a* *t* *u* *s* × ♂*E*.*l* *a* *n* *c* *e* *o* *l* *a* *t* *u*), which, despite being genetically engineered for higher bacterial resistance, exhibited a mortality rate of 29% within 10 days of infection [26]. Similarly, *V. alginolyticus* infections cause mortality in larvae and juveniles within 1–2 weeks postinfection [27, 28]. The impact of these outbreaks extends beyond grouper fish, significantly affecting other aquatic organisms in farmed marine industries. In China, a major *V. alginolyticus* outbreak in 2017 led to mass infections in farmed fish and shellfish, causing tissue necrosis, skin ulceration, gastroenteritis, and inflammatory reactions in aquatic animals [7, 29–32]. The widespread impact of *V. alginolyticus* and concerns over antibiotic resistance, alternative nonantibiotic treatment methods are needed—particularly those not restricted in many countries [33]. One promising approach is bacteriophage (phage) therapy.

Bacteriophage (phage) therapy has demonstrated potential antibacterial activity against specific pathogenic bacteria in humans [34], animals [35], and aquaculture [36]. Additionally, phage therapy has been reported to effectively inhibit *Lactococcus garvieae* [37], *Pseudomonas plecoglossicida* [5], *V. harveyi* [38], and *P. aeruginosa* [39]. In studies on *V. alginolyticus*, phage therapy utilizing the VP01 and pVa‐21 bacterial strains has shown promising antibacterial activity by reducing *V. alginolyticus* growth through infection and subsequent lysis of target cells [40, 41]. These phage strains have demonstrated stability under marine‐mimicking environmental conditions (35°C, pH 7.5–8.4) while effectively inhibiting *V. alginolyticus*.

To the best of our knowledge, no prior studies have reported on the ValKK1‐20 strain. However, we refer to significant findings on the previously studied VP01 and pVa‐21 bacterial strains. Based on these prior reports, we provide data on the characteristics of the ValKK1‐20 strain, a lytic phage with high specificity and effectiveness in targeting and eliminating *V. alginolyticus*.

## 2. Materials and Methods

### 2.1. Isolation of *V. alginolyticus* Phage

*V. alginolyticus* ATCC (American Type Culture Collection) 17749 acquired from ATCC was used as the target bacterium for phage isolation. Briefly, 30 *μ*L glycerol stock of *V. alginolyticus* ATCC 17749 was revived in a double strength (2X) tryptic soy broth (TSB) supplemented with 2% (*v*/*v*) sodium chloride and 0.01 M calcium chloride, at 28°C until the OD_600_ = 1.0 was achieved. Then, equal volume of sand suspension (0.1 g mL^−1^) collected from a beach in Kota Kinabalu, Malaysia (N 06° 02.270 ^′^, E 116°06.710) was mixed with the bacterial suspension and further incubated at 28°C for 24 h. The bacterial cells were removed and the supernatant was mixed with chloroform to final concentration of 40 *μ*L mL^−1^ before centrifugation at 3000 × g for 10 min. The presence of phage was evaluated by using the double layer method following Lal et al. [42].

### 2.2. Host Specificity Test

The host specificity test was done using the spot inoculation method described by Lal et al. [1, 42] on different bacterial species tested in this study "V. alginolyticus ATCC 17749, V. anguillarum ATCC 19264, V. harveyi ATCC 35084, V. parahaemolyticus ATCC 17802". For accuracy, each test was conducted in triplicates.

### 2.3. Molecular Characterization Using Random Amplified Polymorphic DNA (RAPD)

RAPD was used for strain differentiation between phage. The phage genomic DNA was extracted following the method described by Barrangou et al. [43] and Vinod et al. [38] with a few modifications. Fifteen milliliters of each phage lysate was precipitated using polyethylene glycol (PEG)‐sodium chloride (NaCl) solution to a final concentration of 10% PEG: 1 M NaCl. The mixture was incubated at 4°C for 24 h. The phage particles were precipitated by centrifugation at 3000 × g for 20 min. Then, the phage pellet was rinsed once using SM buffer (5.8 g L^−1^ NaCl, 2.0 g L^−1^ MgSO_4_, 50 mL L^−1^ 1 M Tris pH 7.5, 5 mL L^−1^ of presterilized 2% gelatin) and finally suspended in 150 *μ*L of the same buffer. The phage suspension was treated with 1 U of DNase I and RNase A for 30 min at 37°C. After that, the phage suspension was added with 30 *μ*L of SDS solution (10%) and 5 *μ*L of proteinase K (0.001 g mL^−1^) in 600 uL total volume. The suspension was mixed thoroughly and incubated at 37°C for 1 h. After incubation, 80 *μ*L CTAB‐NaCl and 100 *μ*L NaCl 5 M were added and incubated further at 65°C for 10 min. After cooling to room temperature, 500 *μ*L phenol:chloroform:isoamylalcohol (25:24:1) solution was added into the suspension and mixed by inversion. Subsequently, the suspension was separated by centrifugation at 10,000 × g for 5 min. Then, about 500 *μ*L of the aqueous layer was added with an equal volume of chloroform and separated by centrifugation at 16,000 × g for 10 min. The aqueous layer was transferred to a sterile tube and gently mixed with an equal volume of iced‐cold isopropanol until DNA precipitated. The DNA was recovered by centrifugation at 16,000 × g for 10 min. The supernatant was discarded and the DNA pellet was washed using 75% ethanol. Finally, the DNA was briefly air dried and dissolved in 1X TE buffer.

The RAPD analysis was conducted using primer (GTG)_5_: 5 ^′^‐ GTGGTGGTGGTGGTG‐3 ^′^ [44]. The PCR reaction was prepared in 25 *μ*L final volume containing 1X PCR Buffer, 1.7 mM MgCl_2_, 200 *μ*M deoxynucleotide triphosphate (dNTPS), 0.8 *μ*M primer, 1 Unit *Taq* DNA polymerase (Promega), and 50 ng of phage DNA. The PCR reaction was conducted in a thermal cycler (Applied Biosystems Veriti, California) with an initial denaturation step (95°C, 3 min), 45 cycles of denaturation (95°C, 40 s), annealing (38°C, 1 min), and extension (72°C, 1.5 min), and a final extension (72°C, 10 min). The PCR amplicons were separated on 3% agarose gel electrophoresis and visualized using a Gel Documentation System (Villber Lourmat, Germany).

### 2.4. ValKK3 Genomic DNA Extraction and Characterization

The 100 *μ*L of treated bacteriophage particle was topped up to 250 *μ*L total volume in a 1.5 mL microfuge tube. Then, 250 *μ*L buffer ATL and 20 *μ*L Proteinase K were added and mixed for 20 s using a biomixer and incubated at 56°C for 1 h. The tube was cooled at room temperature before adding 4 *μ*L RNase A (100 mg mL^−1^). The tube was further incubated at room temperature for 2 min. After incubation, about 500 *μ*L of buffer AL was added and the tube was vortexed for 15 s using a biomixer. Then, about 200 *μ*L of ethanol (96%–100%) was added. The tube was centrifuged at 6000 × g for 1 min. The spin column was transferred into a new collection tube and the flow‐through was discarded. Then, 500 *μ*L buffer AW1 was added into the spin column and the tube was centrifuged at 6000 × g for 1 min. The flow‐through was discarded and the spin column was transferred into a new collection tube. A 500‐*μ*L buffer AW2 was added and the tube was centrifuged at 20,000 × g for 3 min to dry the spin column. The spin column with bacteriophage DNA was placed into a sterile 1.5‐mL microfuge tube. A 50 *μ*L of nuclease‐free water was added onto the spin column membrane. The spin column was incubated at room temperature for 1 min and the tube was centrifuged for 1 min at 6000 × g. The spin column was discarded and the DNA was stored at −20°C until further use.

The bacteriophage DNA was evaluated using electrophoresis and spectrophotometer. The DNA was run in 1.5% agarose gel to determine the successful extraction of the DNA. The concentration and purity of the DNA was calculated using UV spectrophotometer. The DNA was accepted for sequencing if the DNA was present in the gel, amount of DNA was at least 500 ng in 30‐*μ*L buffer and the purity of the DNA at A260/A280 = 1.8–2.0 (Service provider: AIT Biotech Ptd. Ltd.). The DNA library construction, sequencing and sequence assembly was done by the sequencing service provider (AITBiotech Ptd. Ltd). The DNA libraries were prepared using the Illumina Nextera XT kit according to manufacturer′s specifications. The standard 1 ng of DNA was utilized for tagmentation. The libraries were normalized using beads provided in the Nextera XT kit and pooled in equal ratios. The samples were dual barcoded for multiplexing on the MiSeq instrument. The DNA libraries were added to the MiSeq sequencing reagent cartridge for on‐broad cluster generation and sequencing. The paired 250 bp reads were generated and demultiplexed corresponding to barcodes. The FASQ files were generated on board of MiSeq Instrument. The sequence assembly was performed using de novo assembly. The de novo assembly was performed using paired FASQ files and Velvet assembly software Version 1.1 (Zerbino, European Bioinformatics Institute, United Kingdom). The optimum k‐mersize for assembly was determined using Velvet‐Optimizer Version 2.2.5. The assembly producing the lowest number of contigs with the largest n50 value was chosen for further assembly into single contig.

The putative functions of the ORFs were analyzed by blastn for nucleotide sequence and PSI‐BLAST for peptide sequence [45] against a nonredundant nucleotide and protein database. The ORF was assigned to its function based on the homology searches with blastn and PSI‐BLAST, where the score was > 50 and the e‐value was < 1.0xE‐3 [46]. The additional protein characteristics were predicted using proteomic tools InterPro [47], ScanProsite [48], Pfam [49], and NCBI Conserved Domain Database [50]. Similar to the online software in the previous paragraph, the parameter was set to default. The website of each protein tool was listed in Table 1. Two regions of gene clusters related to DNA replication and regulatory genes and structural protein genes were identified. The lysogeny signature was determined from the protein identification. Meanwhile, the virulence signature from the bacteriophage genome was also determined using two bioinformatics software packages, VFDB [51] and MvirDB [52].

**Table 1:** Online protein sequence analysis tools utilized for the characterization of the protein sequences.

Protein tool	Website
blastn	http://www.ncbi.nlm.nih.gov/blast/Blast.cgi?PROGRAM *=blastn&PAGE_TYPE=BlastSearch&LINK_LOC=blasthome*
PSI‐BLAST	http://www.ncbi.nlm.nih.gov/blast/Blast.cgi?CMD=Web%26PAGE *=Proteins&PROGRAM=blastp&RUN_PSIBLAST=on*
InterPro	http://www.ebi.ac.uk/interpro/
ScanProsite	http://prosite.expasy.org/scanprosite/
Pfam	http://pfam.xfam.org/
NCBI Conserved Domain Database	http://www.ncbi.nlm.nih.gov/Structure/cdd/wrpsb.cgi

### 2.5. Morphological Characteristic, Adsorption Assay, One Step Growth, Stability Test and Coculture of Phage

Based on the result from Section 2.3, one representative strain is randomly selected for further characterization. Morphological characterization, adsorption assay, one step assay, phage stability at different temperatures (40°C, 50°C, 60°C, 70°C, 80°C, 90°C, and 100°C), phage stability at different pH (2, 3, 4, 5, 6, 7, 8, and 9), phage stability at different bile salt concentrations (5000, 6000, 7000, 8000, and 9000 ppm) and in vitro coculture tests were done following Lal et al. [42]. Each assay was conducted in triplicate except for the morphological characterization.

## 3. Results

### 3.1. *V. alginolyticus* ATCC 17749 Phage

**Figure 1:**
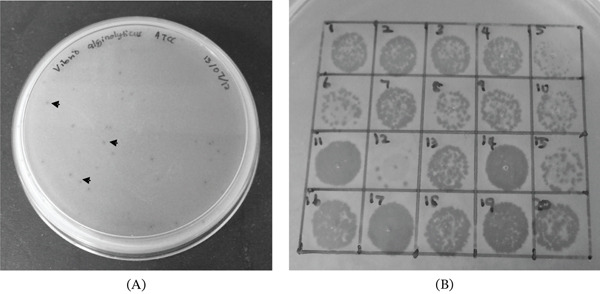
Isolation and confirmation of *Vibrio alginolyticus* phages from seawater samples. (A) Plaque (arrows) forms on *V. alginolyticus* lawn. (B) Plaque confirmation (1 = ValKK1, 2 = ValKK2, 3 = ValKK3, 4 = ValKK4, 5 = ValKK5, 6 = ValKK6, 7 = ValKK7, 8 = ValKK8, 9 = ValKK9, 10 = ValKK10, 11 = ValKK11, 12 = ValKK12, 13 = ValKK13, 14 = ValKK14, 15 = ValKK15, 16 = ValKK16, 17 = ValKK17, 18 = ValKK18, 19 = ValKK19, 20 = ValKK20).

*V. alginolyticus* ATCC 17749 phages were successfully isolated. The plaques are as shown in Figure 1A. The presence of phage was ascertained when formation of clearing zone was evident on freshly prepared bacterial lawn (Figure 1B). About 20 phage isolates were recovered and designated as ValKK1 to ValKK20.

### 3.2. Host Specificity Assay

All phage isolates were selectively lytic to *V. alginolyticus* ATCC 17749 and unable to lyse other bacterial species used in this study (Table 2).

**Table 2:** Host range specificity of *Vibrio alginolyticus* phage isolates (ValKK1‐20) against four different *Vibrio* species.

Host ^∗^	Phage isolates																			
	ValKK																			
	1	2	3	4	5	6	7	8	9	10	11	12	13	14	15	16	17	18	19	20
VALATCC	+	+	+	+	+	+	+	+	+	+	+	+	+	+	+	+	+	+	+	+
VANATCC	−	−	−	−	−	−	−	−	−	−	−	−	−	−	−	−	−	−	−	−
VHATCC	−	−	−	−	−	−	−	−	−	−	−	−	−	−	−	−	−	−	−	−
VPATCC	−	−	−	−	−	−	−	−	−	−	−	−	−	−	−	−	−	−	−	−

*Note:* Asterisk (∗) denotes + = positive; − = negative; VALATCC = *V. alginolyticus* ATCC 17749; VANATCC = *V. anguillarum* ATCC 19264; VHATCC = *V. harveyi* ATCC 35084; VPATCC = *V. parahaemolyticus* ATCC 17802.

### 3.3. Molecular Characterization of Phage

**Figure 2:**
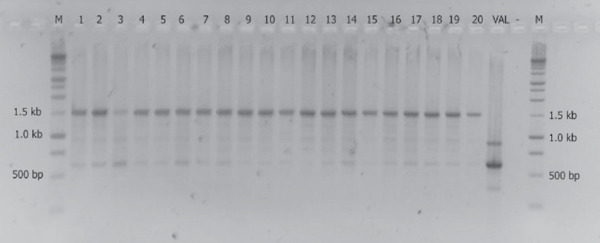
Genetic fingerprinting of *Vibrio alginolyticus* phage isolates (ValKK1‐20) by random amplified polymorphic DNA (RAPD) analysis using (GTG)5 primers. Lane *M* = 1.5 kb DNA ladder, Lane 1–Lane 20 = ValKK1–20, Lane VAL = *V. alginolyticus* genomic DNA, Lane − = sterile distilled water.

The result of RAPD analysis of ValKK1‐20 showed that all phages have identical RAPD pattern (Figure 2). Hence, a phage isolate designated as ValKK3 was randomly selected for further analyses in this study.

### 3.4. ValKK3 Genomic DNA

The size of VALKK3 genome was estimated to have 248,088 bp with *G* + *C* content around 41.2%. The analysis predicted 390 open reading frames, which are numbered consecutively from ORF1 to ORF390 (Table S1). The complete genome sequence has been deposited in the GenBank database under accession number KP671755.

### 3.5. Putative Functional Protein of ValKK3 Genome

**Figure 3:**
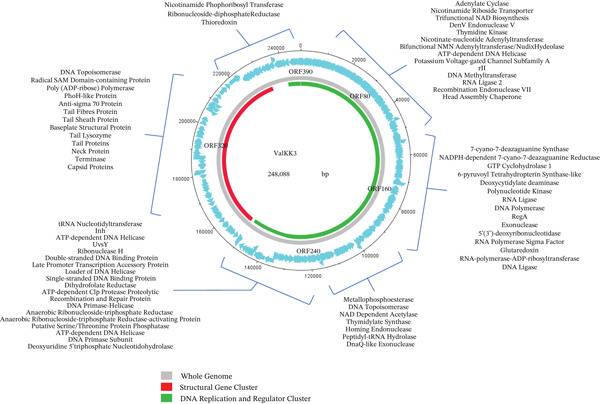
Circular genome map of *Vibrio alginolyticus* phage ValKK3. Predicted open reading frames (ORFs) are represented by colored arrows indicating the direction of transcription. Genes are color‐coded based on predicted functions: Grey represents whole genome, red indicates structural gene, and green indicates DNA replication and regulatory genes.

The putative functional protein in ValKK3 genome was identified using blastn, PSI‐BLAST, Pfam, ScanProsite, NCBI Conserve Domain Database and InterPro (Table S2). Based on the result, the function of predicted 119 of 390 CDSs (coding sequences) could be assigned based on the analyses. The first region of gene cluster which possibly belongs to the DNA replication and regulator cluster covered the region between ORF7 to ORF280 and ORF352 to ORF381. The genome of ValKK3 was presented schematically with predicted CDSs indicated by arrows; the direction of each arrow indicated the direction of the transcription (Figure 3).

The predicted CDSs of ValKK3 represent 92.4% of the total genome. About 59 genes are transcribed in the rightward direction, whereas 331 genes are in the leftward direction. The genes varied from 111 bp (ORF268) to 4200 bp (ORF349). Most of the predicted 375 CDSs start with the AUG codon, whereas 9 and 6 CDSs predicted start with GUG and UUG codons, respectively. At the end of the predicted CDSs in VALKK3, there are 262 UAA codons, 89 UGA codons, and 39 UAG codons as stop codons. The ValKK3 predicted CDSs which fall on the DNA replication and regulator genes are ORF7 (adenylate cyclase), ORF29 (nicotinamide riboside transporter), ORF33 (trifunctional NAD biosynthesis), ORF40 (DenV endonuclease V), ORF61 (thymidine kinase), ORF97 (nicotinate‐nucleotide adenylyltransferase), ORF100 (bifunctional NMN adenylyltransferase/nudix hydrolase), ORF106 (ATP‐dependent DNA helicase), ORF114 (potassium voltage‐gated channel subfamily A), ORF117 (protein rIIB), ORF118 (protein rIIA), ORF124 (DNA methyltransferase), ORF127 (RNA ligase 2), ORF129 (recombination endonuclease VII), ORF131 (head assembly chaperone), ORF136 (7‐cyano‐7‐deazaguanine synthase), ORF137 (NADPH‐dependent 7‐cyano‐7‐deazaguanine reductase), ORF139 (GTP cyclohydrolase 1), ORF140 (6‐pyruvoyl tetrahydropterin synthase‐like protein), ORF141 (deoxycytidylate deaminase), ORF170 (polynucleotide kinase), ORF176 (RNA ligase), ORF178 (DNA polymerase), ORF180 (RegA protein), ORF181 (DNA polymerase accessory protein 62), ORF182 (DNA polymerase accessory protein 44), ORF183 (DNA polymerase processivity component), ORF186 (exonuclease Subunit 2), ORF189 (exonuclease Subunit 1), ORF190 (putative 5 ^′^(3 ^′^)‐deoxyribonucleotidase), ORF197 (RNA polymerase sigma factor), ORF199 (glutaredoxin), ORF200 (SprT‐like protein), ORF202 (RNA polymerase‐ADP‐ribosyltransferase), ORF204 (DNA ligase), ORF212 (metallophosphoesterase), ORF218 (DNA topoisomerase large subunit), ORF219 (NAD‐dependent deacetylase), ORF230 (thymidylate synthase), ORF233 (peptidyl‐tRNA hydrolase), ORF234 (homing endonuclease), ORF239 (DnaQ‐like exonuclease), ORF241 (deoxyuridine 5 ^′^‐triphosphate nucleotidohydrolase), ORF242 (DNA primase subunit), ORF244 (ATP‐dependent DNA helicase UvsW), ORF246 (putative serine/threonine protein phosphatase), ORF247 (anaerobic ribonucleoside‐triphosphate reductase‐activating protein), ORF250 (anaerobic ribonucleoside‐triphosphate reductase), ORF252 (DNA primase‐helicase subunit), ORF254 (recombination and repair protein), ORF255 (ATP‐dependent Clp protease proteolytic Subunit 2), ORF256 (dihydrofolate reductase), ORF257 (single‐stranded DNA‐binding protein), ORF258 (loader of DNA helicase), ORF259 (late promoter transcription accessory protein), ORF260 (double‐stranded DNA‐binding protein), ORF261 (ribonuclease H), ORF264 (UvsY recombination, repair and single‐stranded DNA binding protein), ORF269 (ATP‐dependent DNA helicase), ORF279 (Inh), ORF280 (tRNA nucleotidyltransferase), ORF352 (antisigma70 protein), ORF356 (PhoH‐like protein), ORF358 (poly(ADP‐ribose) polymerase), ORF364 (radical SAM domain‐containing protein), ORF366 (DNA topoisomerase medium subunit), ORF367 (thioredoxin), ORF370 (thioredoxin), ORF371 (ribonucleoside‐diphosphate reductase 1 subunit beta), ORF372 (ribonucleoside‐diphosphate reductase subunit alpha), and ORF381 (nicotinamide phosphoribosyl transferase).

### 3.6. Lysogeny and Virulence Factor

The lysogeny related gene was determined from the predicted protein function of both ValKK3 genomes. The result showed that the lysogeny related protein was absent in the genome. Meanwhile, the virulence signature from the genome was determined using VFBD and MvirDB. The search to VFBD database showed low significant homology. Some of the protein sequences showed no hit to the database. However, the hypothetical protein ORF231 showed low homology to the pilin PilE protein (*Neisseria meningitides*). Similar to VFBD, the MvirDB database showed that the hypothetical proteins have no significant similarity to the sequences in the database.

### 3.7. Morphology of ValKK3

**Figure 4:**
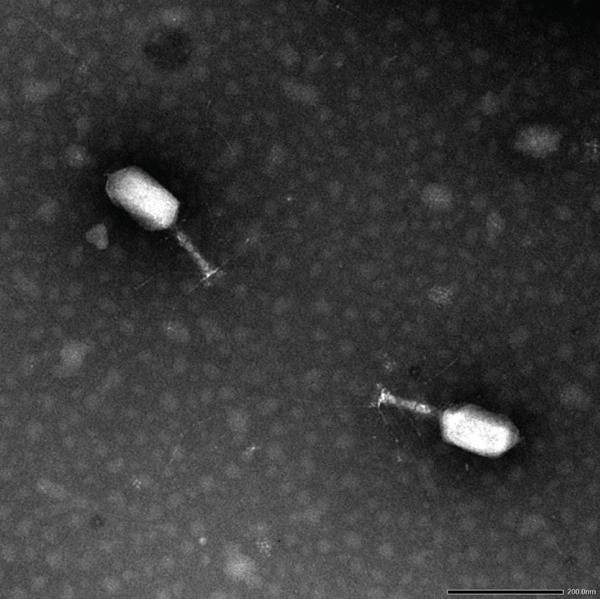
Transmission electron microscopy (TEM) morphology of *Vibrio alginolyticus* phage ValKK3. Bars = 200 nm.

ValKK3 was classified under the Order Caudovirales, family Myoviridae because it was a tailed phage with a tail sheath and a long tail fiber (Figure 4). It has a slightly elongated head based on its dimensions (Table 3) and resembled T4‐like Myoviridae.

**Table 3:** Morphometric dimensions and structural features of phage ValKK3 determined by TEM.

Head length (nm)	Head diameter (nm)	Tail length (nm)	Tail sheath presence	Tail fiber
140.3 ± 3.6	78.8 ± 2.2	122.3 ± 2.9	present	long

### 3.8. Adsorption Assay and One Step Growth

**Figure 5:**
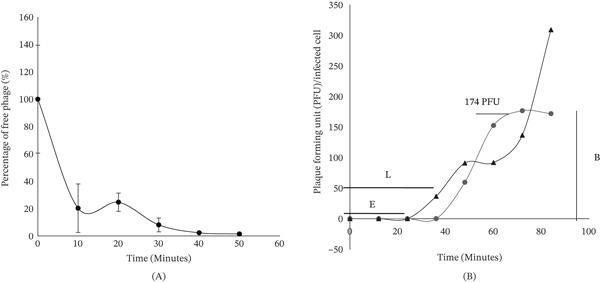
Infection kinetics of *Vibrio alginolyticus* phage ValKK3 against *V. alginolyticus* ATCC 17749TM. Panel (A) shows the adsorption rate of phages to host cells, whereas Panel (B) presents the one‐step growth curve used to determine critical replication parameters. All experiments were performed in triplicate, and data points represent mean values with error bars indicating standard deviation (SD). The number of PFU per infected cell in untreated culture (●) and chloroform‐treated culture (▲) is also shown. The burst size, latent period, and eclipse are indicated as B, L, and E, respectively.

In the adsorption analysis, ValKK3 exhibited two adsorption phases: rapid and slow adsorptions. The rapid adsorption occurred within 10 min, where almost 80% of the phages adsorbed into the host bacteria (Figure 5A). After 10 min, the slow adsorption process took place. The number of free phages was approximately below 20% within 40 min. Meanwhile, one‐step growth analysis (Figure 5B) revealed that the latency and eclipse periods of ValKK3 were at 48 and 36 min, respectively. It has an estimated burst size of 174 pfu/cell at 28°C.

### 3.9. Temperature, pH, and Bile Salt Tolerance

**Figure 6:**
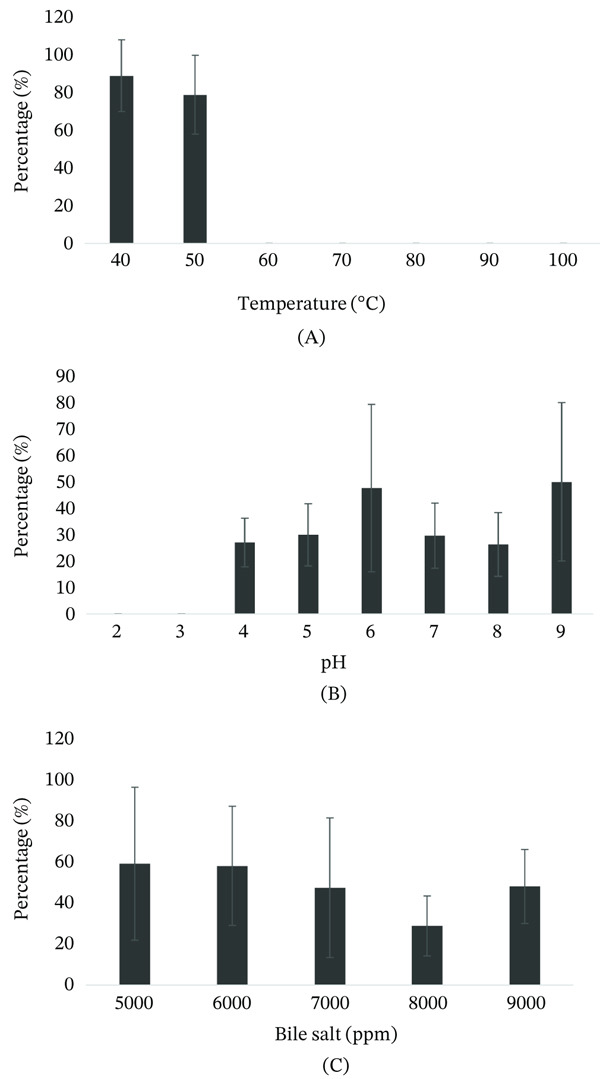
Physical and chemical stability profile of *Vibrio alginolyticus* phage ValKK3. Phage suspensions were exposed to different (A) temperatures, (B) pH levels, and (C) bile salt concentrations. The percentage of surviving phages after exposure was indicated in the graph. All experiments were performed in triplicate, and data points represent mean values with error bars indicating standard deviation (SD).

ValKK3 was stable at 40°C but began to lose activity at 50°C to 80% following heating for 60 min. The lytic activity disappeared entirely when heated at more than 60°C for 1 h (Figure 6A). The lytic activity of ValKK3 can be measured after incubation at pH 4 to pH 9, but disappeared completely at pH 2 and pH 3 (Figure 6B). ValKK3 activities decreased to 20%–40% after 24 h incubation. Meanwhile, the lytic activity of ValKK3 can be detected despite exposure to bile salt concentrations from 5000 to 9000 ppm (Figure 6C).

### 3.10. *In Vitro* Coculture Test

**Figure 7:**
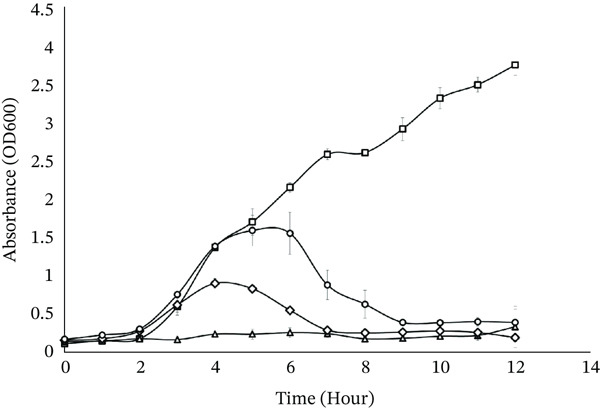
Coculture of ValKK3 phage and *Vibrio alginolyticus* ATCC 17749 at multiplicities of infection (MOI) 0 (), 0.01 (), 1 (), and 100 (). This figure presents the bacterial growth curve of *V. alginolyticus* ATCC 17749 over 12 h when cocultured with phage ValKK3 at MOI. A decrease or suppression of OD₆₀₀ over time indicates phage‐induced bacterial lysis. The control culture (MOI 0) represents bacterial growth in the absence of phage. All experiments were performed in triplicate, and data points represent mean values with error bars indicating standard deviation (SD).

The ability of ValKK3 to lyse its bacterial host was evident. The OD_600_ values of the control cultures (MOI 0) increased during the incubation. In contrast, the OD_600_ values of the bacterial suspension reduced when treated with different MOIs (0.01, 1, and 100) within 12 h period. The bactericidal effect of ValKK3 to *V. alginolyticus* ATCC 17749TM was evident in every MOI (Figure 7). However, at lower MOIs (1 and 0.01), the bacterial growth was only retarded at the fourth and sixth hours after infection, respectively.

## 4. Discussion

Usage of phage for controlling bacterial pathogens is widely accepted. One of the main characteristics is the virulence of the phage. The ValKK3 was virulent and specific to *V. alginolyticus*. This finding was comparable to the *V. alginolyticus* VEN [2]. Although narrow host range phage has limited application in therapy, the high specificity assures the phage is harmless to other bacterial flora [53].

The identical RAPD pattern of the phage isolates in this study indicated that they may belong to the same group. This is in agreement with Hatfull [54] where they noticed phages infecting similar bacterial hosts shared genetic similarity. On the basis of morphological characteristics, ValKK3 is resembled to *Vibrio* phage KVP40 [55] and *Vibrio* phage *φ*pp2 [56]. These two *Vibrio* phages, KVP40 and *φ*pp2, are both have elongated heads with dimensions slightly varied at 90–95 nm wide: 150–160 nm long and 70 nm wide; 140 nm long, respectively. Such characteristics allowed ValKK3 to be classified under the T4‐like phage genus in Myoviridae family and may belong to the giant vibriophage group [56].

The morphological characteristics of ValKK3 was further validated by genome sequencing. The ValKK3 genome (Acc. No.: KP671755; 248,088 bp; 41.2% GC content) is similar to the KVP40 genome (Acc. No.: AY283928; 244,856 bp; 42.6% GC content) and *φ*pp2 genome (Acc. No.: JN849462; 246,421 bp; 42.7% GC content). This makes the ValKK3 belong to the T4‐like phage genus in Myoviridae family and giant vibriophage group (Lin and Lin 2012) with even larger genome size. The blast search revealed that ValKK3 was highly homologous to *Vibrio* phage VH7D (KC131129) infecting *V. harveyi* and *Vibrio* phage KVP40 (AY283928). This result revealed that this T4‐like group was extensively spread in marine environment with the ability to infect different *Vibrio* species. The lysogeny control gene was not identified from both sequences. The T4 phage was categorized as nonlysogenic type phage [57]. The absence of lysogeny related gene correspond to qualify ValKK3 as potential phage therapy candidates since lysogenic phages can lead to virulence enhancement in bacteria [58]. The result of toxin search revealed that one CDS (ORF231) exhibited hit to the toxin database. The CDS showed similarity to the pilin gene from bacteria. However, it was difficult to assure that this gene could contribute to the virulence enhancement in *Vibrio*. This is due to the fact that ValKK3 missing the properties to mediate gene transfer. Furthermore, this gene might not involve in virulence enhancement but more related to bacteriophage attachment to host [59].

The adsorption of the ValKK3 was relatively fast (more than 80% after 10 min) and comparable with *V. parahaemolyticus* phage PW2, Phumkhachorn and Rattanachaikunsopon [60] and *V. parahaemolyticus* phage VpKK5 [42]. However, it differed greatly from *V. harveyi* phage VhKM4 [1]. However, phage adsorption is known to be dependent on various conditions [61]. The ValKK3 exhibited a higher burst size than the фAs51 and фA318 [62]. The shorter latent period and large burst size imply that the phages replicate more quickly so that new virus particles can be released more efficiently [63]. Such characteristics are fundamental requirements in phage therapy [64].

Environmental conditions such as temperature, pH, and bile content are known to affect the survival of phages [65]. In this study, we demonstrated that ValKK3 was viable at 40°C but began to decline at 50°C and was completely inactivated at temperatures over 60°C. It was also inactivated at low pH, but could withstand high bile salt content. Such observation was similar to vibriophage VpKK5 [42]. However, it was different from the studies of Phumkhachorn and Rattanachaikunsopon [60] and Krasowska et al. [66]. Nevertheless, no information on the stability of T4‐like phage was reported previously. Therefore, to the best of our knowledge, the information on phage stability of this phage group of myovirids is only reported in this study. ValKK3 was shown to suppress the growth of *V. alginolyticus* even at low levels of MOI. Unlike VpKK5 [42], the ValKK3 exhibited strong bacteriolytic activity with no emergence of phage‐resistant bacteria was observed. The limitation of this research lies in its focus on in vitro study on *V. alginolyticus* towards changes in pH, temperature, and bile salts. Further studies should investigate the potential of other *V. alginolyticus* isolates with stable phages in marine water and UV stability.

## 5. Conclusion

ValKK3 phage shows a promising biocontrol agent against *V. alginolyticus.* This is due to the phage being genetically identical to the bacterial host under the T4‐like phage genus, with the ability to infect different *Vibrio* species, including *V. alginolyticus.* Additionally, the ValKK3 strain has strong adaptability to extreme conditions, which helps it fight *V. alginolyticus* from different sources. This strain remains viable at temperatures below 40°C and shows high tolerance to pH changes and bile. In conclusion, the ValKK3 bacterial strain has the potential to be used as a phage treatment for vibriosis in the future.

## Author Contributions

M.T.M.L. and J.R. proposed, designed, and supervised the study. E.J.J., G.R., and M.T.M.L. reviewed the literature, carried out the study, interpreted the data, and wrote the draft of the article. M.T.M.L., R.O., N.A.K., M.S., I.B.B.S., A.M.A., and J.R. reviewed and edited the article.

## Funding

This study was supported by Ministry of Higher Education, Malaysia (10.13039/501100003093, HIC2403 FRG0338‐STWN‐1/2013).

## Disclosure

All authors approved the final manuscript.

## Ethics Statement

Ethical approval is not required for the type of research, as it has no involvement of human/animal subjects.

## Conflicts of Interest

The authors declare no conflicts of interest.

## Supporting information


**Supporting Information** Additional supporting information can be found online in the Supporting Information section. All data are included in Supporting Information for Table S1 and S2. All data used in the current research are available from the corresponding author on reasonable request.

## Data Availability

The data that support the findings of this study are available from the corresponding author upon reasonable request.
