# Supplementary material for: Characterization of Giant Myoviridae With Therapeutic Potential Against Grouper Pathogen, Vibrio alginolyticus
Source: Int J Microbiol. 2026 May 5;2026:3932069. doi: 10.1155/ijm/3932069 (PMC13142089; doi:10.1155/ijm/3932069)
Supplement: Supplementary file 1 — Supporting Information Additional supporting information can be found online in the Supporting Information section. All data are included in Supporting Information for Table S1 and S2. All data used in the current research are available from the corresponding author on reasonable request. [file IJM-2026-3932069-s001.docx]

Table S1: Bacteriophage ValKK3 open reading frames

| ORF Name | Strand | Start | End | Nucleotide Length (bp) | Deduced Peptide Length (aa) | Start Codon | Stop Codon |
| --- | --- | --- | --- | --- | --- | --- | --- |
| ORF1 | – | 671 | 324 | 348 | 115 | AUG | UAA |
| ORF2 | – | 1004 | 675 | 330 | 109 | AUG | UAG |
| ORF3 | – | 1477 | 1127 | 351 | 116 | AUG | UAA |
| ORF4 | – | 1706 | 1470 | 237 | 78 | AUG | UAA |
| ORF5 | – | 2239 | 1706 | 534 | 177 | AUG | UGA |
| ORF6 | – | 2722 | 2240 | 483 | 160 | AUG | UAA |
| ORF7 | – | 3546 | 2722 | 825 | 274 | AUG | UAA |
| ORF8 | – | 3833 | 3546 | 288 | 95 | AUG | UAA |
| ORF9 | – | 4090 | 3833 | 258 | 85 | AUG | UAA |
| ORF10 | – | 4545 | 4093 | 453 | 150 | AUG | UAA |
| ORF11 | – | 4864 | 4556 | 309 | 102 | AUG | UAA |
| ORF12 | – | 5106 | 4927 | 180 | 59 | AUG | UGA |
| ORF13 | – | 5381 | 5103 | 279 | 92 | AUG | UGA |
| ORF14 | – | 5741 | 5382 | 360 | 119 | AUG | UAG |
| ORF15 | – | 6118 | 5744 | 375 | 124 | AUG | UAA |
| ORF16 | – | 6480 | 6118 | 363 | 120 | AUG | UAA |
| ORF17 | – | 7244 | 6510 | 735 | 244 | AUG | UAG |
| ORF18 | – | 7401 | 7231 | 171 | 56 | AUG | UAA |
| ORF19 | – | 7672 | 7394 | 279 | 92 | GUG | UAA |
| ORF20 | – | 7914 | 7675 | 240 | 79 | AUG | UAG |
| ORF21 | – | 8156 | 7926 | 231 | 76 | AUG | UAA |
| ORF22 | – | 8520 | 8158 | 363 | 120 | AUG | UAA |
| ORF23 | – | 8845 | 8630 | 216 | 71 | AUG | UAA |
| ORF24 | – | 9114 | 8845 | 270 | 89 | AUG | UGA |
| ORF25 | – | 9310 | 9116 | 195 | 64 | AUG | UAA |
| ORF26 | – | 9847 | 9350 | 498 | 165 | AUG | UAG |
| ORF27 | – | 10131 | 9844 | 288 | 95 | AUG | UGA |
| ORF28 | – | 10534 | 10121 | 414 | 137 | AUG | UAA |
| ORF29 | – | 11205 | 10531 | 675 | 224 | AUG | UGA |
| ORF30 | – | 11496 | 11230 | 267 | 88 | AUG | UAA |
| ORF31 | – | 11837 | 11493 | 345 | 114 | AUG | UGA |
| ORF32 | – | 12166 | 11831 | 336 | 111 | AUG | UAA |
| ORF33 | – | 13137 | 12157 | 981 | 326 | AUG | UAA |
| ORF34 | – | 13307 | 13122 | 186 | 61 | AUG | UAA |
| ORF35 | – | 13528 | 13304 | 225 | 74 | AUG | UGA |
| ORF36 | – | 14227 | 13754 | 474 | 157 | GUG | UAA |
| ORF37 | – | 14605 | 14381 | 225 | 74 | AUG | UAA |
| ORF38 | – | 14924 | 14571 | 354 | 117 | AUG | UAA |
| ORF39 | – | 15358 | 14921 | 438 | 145 | AUG | UGA |
| ORF40 | – | 15791 | 15369 | 423 | 140 | AUG | UGA |
| ORF41 | – | 16053 | 15784 | 270 | 89 | AUG | UAG |
| ORF42 | – | 16331 | 16050 | 282 | 93 | AUG | UGA |
| ORF43 | – | 16623 | 16333 | 291 | 96 | AUG | UAA |
| ORF44 | – | 17351 | 16698 | 654 | 217 | AUG | UAA |
| ORF45 | – | 17761 | 17360 | 402 | 133 | AUG | UAG |
| ORF46 | – | 18356 | 17763 | 594 | 197 | AUG | UAA |
| ORF47 | – | 18736 | 18344 | 393 | 130 | AUG | UGA |
| ORF48 | – | 19296 | 18736 | 561 | 186 | AUG | UAA |
| ORF49 | – | 19964 | 19329 | 636 | 211 | AUG | UAA |
| ORF50 | – | 20144 | 19974 | 171 | 56 | AUG | UAA |
| ORF51 | – | 20391 | 20146 | 246 | 81 | AUG | UAA |
| ORF52 | – | 20644 | 20384 | 261 | 86 | AUG | UGA |
| ORF53 | – | 20879 | 20628 | 252 | 83 | AUG | UAA |
| ORF54 | – | 21163 | 20861 | 303 | 100 | AUG | UGA |
| ORF55 | – | 21747 | 21163 | 585 | 194 | AUG | UAA |
| ORF56 | – | 22203 | 21910 | 294 | 97 | AUG | UAA |
| ORF57 | – | 22520 | 22236 | 285 | 94 | AUG | UAA |
| ORF58 | – | 22843 | 22520 | 324 | 107 | AUG | UAA |
| ORF59 | – | 23187 | 22840 | 348 | 115 | AUG | UGA |
| ORF60 | – | 23486 | 23184 | 303 | 100 | AUG | UGA |
| ORF61 | – | 24056 | 23487 | 570 | 189 | AUG | UAG |
| ORF62 | – | 24394 | 24065 | 330 | 109 | AUG | UAA |
| ORF63 | – | 25308 | 24394 | 915 | 304 | GUG | UGA |
| ORF64 | – | 25618 | 25298 | 321 | 106 | AUG | UGA |
| ORF65 | – | 25770 | 25615 | 156 | 51 | AUG | UGA |
| ORF66 | – | 25952 | 25767 | 186 | 61 | AUG | UGA |
| ORF67 | – | 26383 | 25949 | 435 | 144 | AUG | UGA |
| ORF68 | – | 26540 | 26385 | 156 | 51 | AUG | UAA |
| ORF69 | – | 26754 | 26548 | 207 | 68 | AUG | UGA |
| ORF70 | – | 27070 | 26735 | 336 | 111 | AUG | UAG |
| ORF71 | – | 27284 | 27063 | 222 | 73 | AUG | UAA |
| ORF72 | – | 27592 | 27284 | 309 | 102 | AUG | UAA |
| ORF73 | – | 27831 | 27589 | 243 | 80 | AUG | UGA |
| ORF74 | – | 28064 | 27828 | 237 | 78 | AUG | UGA |
| ORF75 | – | 28399 | 28046 | 354 | 117 | AUG | UAA |
| ORF76 | – | 28869 | 28396 | 474 | 157 | AUG | UGA |
| ORF77 | – | 29284 | 28862 | 423 | 140 | AUG | UAG |
| ORF78 | – | 29661 | 29284 | 378 | 125 | AUG | UAA |
| ORF79 | – | 30097 | 29663 | 435 | 144 | AUG | UAA |
| ORF80 | – | 30352 | 30101 | 252 | 83 | AUG | UAA |

| ORF Name | Strand | Start | End | Nucleotide Length (bp) | Deduced Peptide Length (aa) | Start Codon | Stop Codon |
| --- | --- | --- | --- | --- | --- | --- | --- |
| ORF81 | – | 30587 | 30414 | 174 | 57 | AUG | UAA |
| ORF82 | – | 31081 | 30674 | 408 | 135 | AUG | UAA |
| ORF83 | – | 31403 | 31086 | 318 | 105 | AUG | UAA |
| ORF84 | – | 32198 | 31590 | 609 | 202 | AUG | UGA |
| ORF85 | – | 32517 | 32209 | 309 | 102 | AUG | UAA |
| ORF86 | – | 32837 | 32514 | 324 | 107 | AUG | UGA |
| ORF87 | – | 33639 | 33472 | 168 | 55 | AUG | UAA |
| ORF88 | – | 33859 | 33644 | 216 | 71 | AUG | UAA |
| ORF89 | – | 34020 | 33856 | 165 | 54 | AUG | UGA |
| ORF90 | – | 34190 | 34017 | 174 | 57 | AUG | UGA |
| ORF91 | – | 34440 | 34201 | 240 | 79 | AUG | UAA |
| ORF92 | – | 34839 | 34510 | 330 | 109 | AUG | UAA |
| ORF93 | – | 35012 | 34842 | 171 | 56 | AUG | UAA |
| ORF94 | – | 35227 | 35015 | 213 | 70 | AUG | UGA |
| ORF95 | – | 35439 | 35224 | 216 | 71 | AUG | UGA |
| ORF96 | – | 35633 | 35436 | 198 | 65 | AUG | UGA |
| ORF97 | – | 36244 | 35618 | 627 | 208 | AUG | UGA |
| ORF98 | – | 36596 | 36246 | 351 | 116 | AUG | UAA |
| ORF99 | – | 36865 | 36593 | 273 | 90 | AUG | UGA |
| ORF100 | – | 37928 | 36903 | 1026 | 341 | AUG | UAA |
| ORF101 | – | 38513 | 37998 | 516 | 171 | AUG | UAA |
| ORF102 | – | 38979 | 38518 | 462 | 153 | AUG | UAA |
| ORF103 | – | 39792 | 39031 | 762 | 253 | AUG | UAA |
| ORF104 | – | 40036 | 39857 | 180 | 59 | AUG | UAA |
| ORF105 | – | 40356 | 40033 | 324 | 107 | AUG | UGA |
| ORF106 | – | 41700 | 40435 | 1266 | 421 | GUG | UAA |
| ORF107 | – | 42085 | 41711 | 375 | 124 | AUG | UGA |
| ORF108 | – | 42537 | 42082 | 456 | 151 | AUG | UGA |
| ORF109 | – | 42831 | 42580 | 252 | 83 | AUG | UAA |
| ORF110 | – | 43879 | 42866 | 1014 | 337 | AUG | UGA |
| ORF111 | – | 44151 | 43876 | 276 | 91 | AUG | UGA |
| ORF112 | – | 44887 | 45444 | 558 | 185 | AUG | UAA |
| ORF113 | – | 45964 | 45473 | 492 | 163 | AUG | UAA |
| ORF114 | – | 46644 | 45961 | 684 | 227 | AUG | UGA |
| ORF115 | – | 46975 | 46631 | 345 | 114 | AUG | UAA |
| ORF116 | – | 47245 | 46988 | 258 | 85 | AUG | UAA |
| ORF117 | – | 48356 | 47325 | 1032 | 343 | AUG | UAA |
| ORF118 | – | 50418 | 48349 | 2070 | 689 | AUG | UAA |
| ORF119 | – | 50563 | 50426 | 138 | 45 | AUG | UGA |
| ORF120 | – | 50915 | 50619 | 297 | 98 | AUG | UAA |
| ORF121 | – | 51180 | 50908 | 273 | 90 | AUG | UAG |
| ORF122 | – | 51530 | 51174 | 357 | 118 | AUG | UAG |
| ORF123 | – | 51763 | 51548 | 216 | 71 | AUG | UGA |
| ORF124 | – | 52338 | 51760 | 579 | 192 | AUG | UGA |
| ORF125 | – | 52867 | 52340 | 528 | 175 | AUG | UAA |
| ORF126 | – | 53588 | 53037 | 552 | 183 | AUG | UAA |
| ORF127 | – | 54782 | 53775 | 1008 | 335 | AUG | UAA |
| ORF128 | – | 55040 | 54792 | 249 | 82 | AUG | UAA |
| ORF129 | + | 55120 | 55626 | 507 | 168 | AUG | UAG |
| ORF130 | – | 56044 | 55655 | 390 | 129 | AUG | UAA |
| ORF131 | – | 56385 | 56047 | 339 | 112 | AUG | UAA |
| ORF132 | – | 56591 | 56385 | 207 | 68 | AUG | UAA |
| ORF133 | – | 56895 | 56686 | 210 | 69 | UUG | UGA |
| ORF134 | – | 57163 | 56888 | 276 | 91 | AUG | UAA |
| ORF135 | – | 57781 | 57173 | 609 | 202 | AUG | UGA |
| ORF136 | – | 58497 | 57781 | 717 | 238 | AUG | UGA |
| ORF137 | – | 59461 | 58553 | 909 | 302 | AUG | UAA |
| ORF138 | – | 60867 | 59491 | 1377 | 458 | AUG | UAG |
| ORF139 | – | 61539 | 60871 | 669 | 222 | AUG | UAA |
| ORF140 | – | 62539 | 61607 | 933 | 310 | AUG | UAA |
| ORF141 | – | 63046 | 62594 | 453 | 150 | AUG | UAA |
| ORF142 | – | 63363 | 63046 | 318 | 105 | AUG | UAA |
| ORF143 | – | 63724 | 63425 | 300 | 99 | AUG | UAG |
| ORF144 | – | 63931 | 63737 | 195 | 64 | AUG | UAA |
| ORF145 | – | 64939 | 64064 | 876 | 291 | AUG | UAA |
| ORF146 | – | 65861 | 64941 | 921 | 306 | AUG | UAA |
| ORF147 | – | 66314 | 65865 | 450 | 149 | AUG | UAG |
| ORF148 | – | 66604 | 66311 | 294 | 97 | AUG | UGA |
| ORF149 | – | 66892 | 66608 | 285 | 94 | AUG | UAA |
| ORF150 | – | 67187 | 66882 | 306 | 101 | AUG | UAA |
| ORF151 | – | 67396 | 67217 | 180 | 59 | AUG | UAA |
| ORF152 | – | 68817 | 67441 | 1377 | 458 | AUG | UAA |
| ORF153 | – | 69228 | 68947 | 282 | 93 | AUG | UAA |
| ORF154 | – | 69560 | 69225 | 336 | 111 | AUG | UGA |
| ORF155 | – | 69799 | 69560 | 240 | 79 | AUG | UAA |
| ORF156 | – | 70196 | 69804 | 393 | 130 | AUG | UAA |
| ORF157 | – | 70894 | 70199 | 696 | 231 | AUG | UAA |
| ORF158 | + | 70972 | 72027 | 1056 | 351 | AUG | UAA |
| ORF159 | – | 72669 | 72049 | 621 | 206 | AUG | UGA |
| ORF160 | – | 73059 | 72682 | 378 | 125 | AUG | UGA |

| ORF Name | Strand | Start | End | Nucleotide Length (bp) | Deduced Peptide Length (aa) | Start Codon | Stop Codon |
| --- | --- | --- | --- | --- | --- | --- | --- |
| ORF161 | – | 73727 | 73104 | 624 | 207 | AUG | UGA |
| ORF162 | – | 74001 | 73729 | 273 | 90 | AUG | UAA |
| ORF163 | – | 75527 | 74076 | 1452 | 483 | AUG | UAA |
| ORF164 | – | 76000 | 75602 | 399 | 132 | AUG | UAG |
| ORF165 | – | 76184 | 75993 | 192 | 63 | AUG | UGA |
| ORF166 | – | 76407 | 76177 | 231 | 76 | AUG | UAA |
| ORF167 | – | 76574 | 76404 | 171 | 56 | AUG | UGA |
| ORF168 | – | 77090 | 76578 | 513 | 170 | AUG | UGA |
| ORF169 | – | 77641 | 77090 | 552 | 183 | AUG | UAA |
| ORF170 | – | 78569 | 77652 | 918 | 305 | AUG | UAA |
| ORF171 | – | 78811 | 78578 | 234 | 77 | AUG | UAA |
| ORF172 | – | 79109 | 78813 | 297 | 98 | AUG | UAA |
| ORF173 | – | 79518 | 79189 | 330 | 109 | AUG | UAA |
| ORF174 | – | 79861 | 79508 | 354 | 117 | UUG | UAA |
| ORF175 | – | 80203 | 79934 | 270 | 89 | AUG | UAA |
| ORF176 | – | 81349 | 80204 | 1146 | 381 | AUG | UAA |
| ORF177 | – | 81679 | 81413 | 267 | 88 | UUG | UAA |
| ORF178 | – | 84224 | 81672 | 2553 | 850 | AUG | UAA |
| ORF179 | – | 84870 | 84259 | 612 | 203 | AUG | UAA |
| ORF180 | – | 85322 | 84942 | 381 | 126 | AUG | UAA |
| ORF181 | – | 85848 | 85357 | 492 | 163 | AUG | UAA |
| ORF182 | – | 86808 | 85858 | 951 | 316 | AUG | UAA |
| ORF183 | – | 87540 | 86875 | 666 | 221 | AUG | UAA |
| ORF184 | – | 87784 | 87575 | 210 | 69 | AUG | UAA |
| ORF185 | – | 88305 | 87841 | 465 | 154 | AUG | UAG |
| ORF186 | – | 90539 | 88302 | 2238 | 745 | AUG | UGA |
| ORF187 | – | 90828 | 90532 | 297 | 98 | AUG | UAA |
| ORF188 | – | 91048 | 90821 | 228 | 75 | AUG | UAA |
| ORF189 | – | 92091 | 91048 | 1044 | 347 | AUG | UGA |
| ORF190 | – | 92600 | 92088 | 513 | 170 | AUG | UGA |
| ORF191 | – | 93042 | 92578 | 465 | 154 | AUG | UAA |
| ORF192 | – | 93392 | 93042 | 351 | 116 | AUG | UAA |
| ORF193 | – | 93853 | 93392 | 462 | 153 | AUG | UAA |
| ORF194 | – | 94167 | 93901 | 267 | 88 | AUG | UAA |
| ORF195 | – | 94909 | 94262 | 648 | 215 | AUG | UAA |
| ORF196 | – | 96362 | 94917 | 1446 | 481 | AUG | UAG |
| ORF197 | – | 96884 | 96372 | 513 | 170 | AUG | UAA |
| ORF198 | – | 97792 | 96893 | 900 | 299 | AUG | UAA |
| ORF199 | – | 98087 | 97848 | 240 | 79 | AUG | UAA |
| ORF200 | – | 99082 | 98255 | 828 | 275 | AUG | UAA |
| ORF201 | – | 99694 | 99191 | 504 | 167 | AUG | UAA |
| ORF202 | + | 99853 | 101481 | 1629 | 542 | AUG | UGA |
| ORF203 | – | 102398 | 101511 | 888 | 295 | AUG | UAA |
| ORF204 | – | 103802 | 102465 | 1338 | 445 | AUG | UAA |
| ORF205 | – | 103983 | 103780 | 204 | 67 | AUG | UAA |
| ORF206 | – | 104233 | 103934 | 300 | 99 | AUG | UGA |
| ORF207 | – | 104890 | 104297 | 594 | 197 | AUG | UAA |
| ORF208 | – | 105101 | 104904 | 198 | 65 | AUG | UAA |
| ORF209 | – | 105675 | 105166 | 510 | 169 | AUG | UAA |
| ORF210 | – | 105980 | 105735 | 246 | 81 | AUG | UAA |
| ORF211 | – | 106209 | 106021 | 189 | 62 | AUG | UGA |
| ORF212 | – | 106934 | 106206 | 729 | 242 | AUG | UGA |
| ORF213 | – | 107191 | 106922 | 270 | 89 | AUG | UAA |
| ORF214 | – | 107580 | 107188 | 393 | 130 | AUG | UGA |
| ORF215 | – | 107858 | 107592 | 267 | 88 | AUG | UGA |
| ORF216 | – | 108091 | 107876 | 216 | 71 | AUG | UAA |
| ORF217 | – | 108321 | 108091 | 231 | 76 | AUG | UAA |
| ORF218 | – | 110114 | 108318 | 1797 | 598 | AUG | UGA |
| ORF219 | – | 111007 | 110246 | 762 | 253 | UUG | UAG |
| ORF220 | – | 111372 | 111004 | 369 | 122 | AUG | UGA |
| ORF221 | – | 111629 | 111369 | 261 | 86 | AUG | UGA |
| ORF222 | – | 112032 | 111631 | 402 | 133 | AUG | UAA |
| ORF223 | – | 112364 | 112035 | 330 | 109 | AUG | UAA |
| ORF224 | – | 113196 | 112435 | 762 | 253 | AUG | UGA |
| ORF225 | – | 113492 | 113199 | 294 | 97 | AUG | UAG |
| ORF226 | – | 113813 | 113493 | 321 | 106 | AUG | UAA |
| ORF227 | – | 114100 | 113810 | 291 | 96 | AUG | UGA |
| ORF228 | – | 114306 | 114100 | 207 | 68 | AUG | UAA |
| ORF229 | – | 114502 | 114308 | 195 | 64 | GUG | UAA |
| ORF230 | – | 115398 | 114499 | 900 | 299 | AUG | UGA |
| ORF231 | – | 115611 | 115408 | 204 | 67 | AUG | UAA |
| ORF232 | – | 115878 | 115648 | 231 | 76 | AUG | UAA |
| ORF233 | – | 116317 | 115871 | 447 | 148 | AUG | UAA |
| ORF234 | + | 116394 | 117044 | 651 | 216 | AUG | UAA |
| ORF235 | – | 117264 | 117010 | 255 | 84 | AUG | UAA |
| ORF236 | – | 117649 | 117251 | 399 | 132 | AUG | UAG |
| ORF237 | – | 117783 | 117646 | 138 | 45 | AUG | UGA |
| ORF238 | – | 118013 | 117852 | 162 | 53 | AUG | UAA |
| ORF239 | – | 118702 | 118010 | 693 | 230 | AUG | UGA |
| ORF240 | – | 118941 | 118699 | 243 | 80 | AUG | UGA |

| ORF Name | Strand | Start | End | Nucleotide Length (bp) | Deduced Peptide Length (aa) | Start Codon | Stop Codon |
| --- | --- | --- | --- | --- | --- | --- | --- |
| ORF241 | – | 119525 | 118941 | 585 | 194 | AUG | UAA |
| ORF242 | – | 120583 | 119525 | 1059 | 352 | AUG | UAA |
| ORF243 | – | 121143 | 120664 | 480 | 159 | AUG | UAA |
| ORF244 | – | 121967 | 121143 | 825 | 274 | AUG | UAA |
| ORF245 | – | 122816 | 121971 | 846 | 281 | AUG | UAG |
| ORF246 | – | 123405 | 122881 | 525 | 174 | AUG | UAA |
| ORF247 | – | 123881 | 123405 | 477 | 158 | GUG | UAA |
| ORF248 | – | 124464 | 123862 | 603 | 200 | AUG | UAA |
| ORF249 | – | 124882 | 124466 | 417 | 138 | AUG | UAA |
| ORF250 | – | 126835 | 125000 | 1836 | 611 | AUG | UGA |
| ORF251 | – | 127081 | 126836 | 246 | 81 | UUG | UGA |
| ORF252 | – | 128475 | 127075 | 1401 | 466 | AUG | UAG |
| ORF253 | – | 128714 | 128412 | 303 | 100 | AUG | UAA |
| ORF254 | – | 129869 | 128769 | 1101 | 366 | AUG | UAA |
| ORF255 | – | 130652 | 129936 | 717 | 238 | AUG | UAA |
| ORF256 | – | 131194 | 130649 | 546 | 181 | AUG | UGA |
| ORF257 | – | 132170 | 131244 | 927 | 308 | AUG | UAA |
| ORF258 | – | 132781 | 132221 | 561 | 186 | AUG | UAA |
| ORF259 | – | 133109 | 132816 | 294 | 97 | AUG | UAG |
| ORF260 | – | 133383 | 133102 | 282 | 93 | AUG | UAA |
| ORF261 | – | 134398 | 133466 | 933 | 310 | AUG | UAA |
| ORF262 | + | 134477 | 138160 | 3684 | 1227 | AUG | UAA |
| ORF263 | + | 138169 | 140862 | 2694 | 897 | AUG | UAA |
| ORF264 | + | 141031 | 141444 | 414 | 137 | AUG | UGA |
| ORF265 | + | 141441 | 141854 | 414 | 137 | AUG | UAA |
| ORF266 | + | 141856 | 142020 | 165 | 54 | AUG | UAA |
| ORF267 | + | 142020 | 142190 | 171 | 56 | AUG | UAA |
| ORF268 | + | 142195 | 142305 | 111 | 36 | AUG | UAA |
| ORF269 | – | 143873 | 142341 | 1533 | 510 | AUG | UAG |
| ORF270 | + | 143938 | 144147 | 210 | 69 | AUG | UAA |
| ORF271 | + | 144157 | 144669 | 513 | 170 | AUG | UAA |
| ORF272 | + | 144673 | 145161 | 489 | 162 | AUG | UAG |
| ORF273 | + | 145228 | 145494 | 267 | 88 | AUG | UAA |
| ORF274 | + | 145510 | 146136 | 627 | 208 | AUG | UAA |
| ORF275 | + | 146215 | 146808 | 594 | 197 | AUG | UAA |
| ORF276 | + | 146811 | 147731 | 921 | 306 | AUG | UAA |
| ORF277 | + | 147904 | 149088 | 1185 | 394 | AUG | UAG |
| ORF278 | – | 149481 | 149146 | 336 | 111 | AUG | UAA |
| ORF279 | – | 149987 | 149490 | 498 | 165 | AUG | UAG |
| ORF280 | + | 150083 | 151180 | 1098 | 365 | AUG | UAA |
| ORF281 | – | 152781 | 151237 | 1545 | 514 | AUG | UAA |
| ORF282 | – | 153700 | 152852 | 849 | 282 | AUG | UAA |
| ORF283 | – | 154374 | 153733 | 642 | 213 | AUG | UAA |
| ORF284 | – | 154868 | 154377 | 492 | 163 | AUG | UAA |
| ORF285 | – | 155036 | 154869 | 168 | 55 | AUG | UAA |
| ORF286 | – | 156600 | 155050 | 1551 | 516 | AUG | UAA |
| ORF287 | – | 157140 | 156640 | 501 | 166 | AUG | UAA |
| ORF288 | – | 159207 | 157192 | 2016 | 671 | AUG | UAA |
| ORF289 | – | 161056 | 159254 | 1803 | 600 | AUG | UAG |
| ORF290 | – | 161564 | 161016 | 549 | 182 | AUG | UAA |
| ORF291 | – | 162223 | 161675 | 549 | 182 | AUG | UAA |
| ORF292 | – | 163235 | 162276 | 960 | 319 | GUG | UAA |
| ORF293 | – | 164186 | 163347 | 840 | 279 | AUG | UAA |
| ORF294 | – | 165119 | 164190 | 930 | 309 | AUG | UAG |
| ORF295 | – | 166812 | 165130 | 1683 | 560 | AUG | UAA |
| ORF296 | – | 168538 | 167111 | 1428 | 475 | AUG | UAA |
| ORF297 | – | 170073 | 168535 | 1539 | 512 | AUG | UGA |
| ORF298 | – | 170759 | 170073 | 687 | 228 | AUG | UAA |
| ORF299 | – | 173005 | 170759 | 2247 | 748 | AUG | UAA |
| ORF300 | – | 173971 | 173015 | 957 | 318 | AUG | UAA |
| ORF301 | – | 175047 | 174025 | 1023 | 340 | GUG | UAA |
| ORF302 | – | 178546 | 175049 | 3498 | 1165 | AUG | UAA |
| ORF303 | – | 180504 | 178546 | 1959 | 652 | AUG | UAA |
| ORF304 | – | 181009 | 180590 | 420 | 139 | AUG | UAA |
| ORF305 | + | 181152 | 182027 | 876 | 291 | AUG | UAA |
| ORF306 | + | 182077 | 182436 | 360 | 119 | AUG | UAA |
| ORF307 | – | 182719 | 182423 | 297 | 98 | AUG | UAG |
| ORF308 | – | 183204 | 182719 | 486 | 161 | AUG | UAA |
| ORF309 | – | 184420 | 183209 | 1212 | 403 | AUG | UAA |
| ORF310 | – | 185697 | 184426 | 1272 | 423 | AUG | UAA |
| ORF311 | – | 186277 | 185699 | 579 | 192 | AUG | UAA |
| ORF312 | – | 187410 | 186274 | 1137 | 378 | AUG | UGA |
| ORF313 | + | 187478 | 187933 | 456 | 151 | AUG | UAA |
| ORF314 | + | 187935 | 188531 | 597 | 198 | AUG | UAA |
| ORF315 | + | 188535 | 189281 | 747 | 248 | AUG | UAA |
| ORF316 | + | 189294 | 190142 | 849 | 282 | AUG | UAA |
| ORF317 | + | 190142 | 190309 | 168 | 55 | AUG | UAA |
| ORF318 | + | 190309 | 192282 | 1974 | 657 | AUG | UAA |
| ORF319 | + | 192282 | 192818 | 537 | 178 | AUG | UAG |
| ORF320 | + | 193049 | 193693 | 645 | 214 | AUG | UGA |

| ORF Name | Strand | Start | End | Nucleotide Length (bp) | Deduced Peptide Length (aa) | Start Codon | Stop Codon |
| --- | --- | --- | --- | --- | --- | --- | --- |
| ORF321 | + | 193698 | 193964 | 267 | 88 | AUG | UAA |
| ORF322 | + | 193964 | 194419 | 456 | 151 | AUG | UGA |
| ORF323 | + | 194412 | 194666 | 255 | 84 | AUG | UAA |
| ORF324 | + | 195976 | 196344 | 369 | 122 | AUG | UGA |
| ORF325 | + | 196503 | 196733 | 231 | 76 | AUG | UAA |
| ORF326 | + | 196991 | 197515 | 525 | 174 | AUG | UAA |
| ORF327 | + | 197547 | 197843 | 297 | 98 | AUG | UAA |
| ORF328 | + | 197991 | 198227 | 237 | 78 | AUG | UAA |
| ORF329 | + | 199181 | 199399 | 219 | 72 | AUG | UAG |
| ORF330 | + | 201182 | 201457 | 216 | 71 | AUG | UAA |
| ORF331 | + | 201522 | 201683 | 162 | 53 | AUG | UAA |
| ORF332 | + | 202878 | 203117 | 240 | 79 | AUG | UGA |
| ORF333 | – | 206212 | 204497 | 1716 | 571 | AUG | UAA |
| ORF334 | – | 206560 | 206222 | 339 | 112 | AUG | UAA |
| ORF335 | + | 206632 | 207009 | 378 | 125 | AUG | UAA |
| ORF336 | + | 207042 | 207248 | 207 | 68 | AUG | UAA |
| ORF337 | + | 207248 | 207481 | 234 | 77 | AUG | UAA |
| ORF338 | + | 207580 | 207792 | 213 | 70 | AUG | UAA |
| ORF339 | – | 208195 | 207824 | 372 | 123 | AUG | UAA |
| ORF340 | + | 208262 | 208609 | 348 | 115 | AUG | UAA |
| ORF341 | + | 208600 | 208965 | 366 | 121 | AUG | UGA |
| ORF342 | + | 208962 | 209297 | 336 | 111 | AUG | UGA |
| ORF343 | + | 209294 | 209608 | 315 | 104 | AUG | UAA |
| ORF344 | + | 209700 | 211136 | 1437 | 478 | AUG | UAA |
| ORF345 | + | 211186 | 211710 | 525 | 174 | AUG | UAG |
| ORF346 | + | 211778 | 213316 | 1539 | 512 | AUG | UAA |
| ORF347 | + | 213328 | 216786 | 3459 | 1152 | AUG | UAA |
| ORF348 | + | 216817 | 218547 | 1731 | 576 | AUG | UAA |
| ORF349 | + | 218618 | 222817 | 4200 | 1399 | AUG | UAA |
| ORF350 | + | 222867 | 223442 | 576 | 191 | AUG | UGA |
| ORF351 | – | 223772 | 223476 | 297 | 98 | AUG | UAA |
| ORF352 | – | 224081 | 223782 | 300 | 99 | AUG | UAA |
| ORF353 | – | 224817 | 224086 | 732 | 243 | AUG | UAA |
| ORF354 | – | 224977 | 224777 | 201 | 66 | AUG | UAG |
| ORF355 | – | 225360 | 225052 | 309 | 102 | AUG | UAA |
| ORF356 | – | 226064 | 225360 | 705 | 234 | AUG | UAA |
| ORF357 | – | 226612 | 226127 | 486 | 161 | AUG | UAG |
| ORF358 | – | 227763 | 226654 | 1110 | 369 | AUG | UAA |
| ORF359 | – | 228113 | 227841 | 273 | 90 | AUG | UAA |
| ORF360 | – | 228390 | 228121 | 270 | 89 | AUG | UGA |
| ORF361 | – | 228682 | 228383 | 300 | 99 | AUG | UAA |
| ORF362 | – | 228827 | 228672 | 156 | 51 | AUG | UAA |
| ORF363 | – | 229150 | 228893 | 258 | 85 | AUG | UAG |
| ORF364 | – | 230072 | 229188 | 885 | 294 | AUG | UAA |
| ORF365 | – | 230323 | 230081 | 243 | 80 | AUG | UAA |
| ORF366 | – | 231609 | 230323 | 1287 | 428 | AUG | UAA |
| ORF367 | – | 232659 | 231655 | 1005 | 334 | AUG | UAA |
| ORF368 | – | 232945 | 232760 | 186 | 61 | AUG | UAA |
| ORF369 | – | 233517 | 232945 | 573 | 190 | AUG | UAA |
| ORF370 | – | 233890 | 233591 | 300 | 99 | UUG | UAA |
| ORF371 | – | 235016 | 233892 | 1125 | 374 | AUG | UAA |
| ORF372 | – | 237251 | 235026 | 2226 | 741 | AUG | UAA |
| ORF373 | – | 237760 | 237284 | 477 | 158 | AUG | UAA |
| ORF374 | – | 237967 | 237764 | 204 | 67 | AUG | UAA |
| ORF375 | + | 238036 | 238266 | 231 | 76 | AUG | UAA |
| ORF376 | – | 238787 | 238281 | 507 | 168 | AUG | UAA |
| ORF377 | – | 239635 | 238916 | 720 | 239 | AUG | UAA |
| ORF378 | – | 239907 | 239647 | 261 | 86 | AUG | UAA |
| ORF379 | – | 241201 | 239972 | 1230 | 409 | AUG | UAA |
| ORF380 | – | 241580 | 241254 | 327 | 108 | AUG | UAA |
| ORF381 | – | 243181 | 241688 | 1494 | 497 | AUG | UAA |
| ORF382 | – | 243745 | 243242 | 504 | 167 | AUG | UAA |
| ORF383 | – | 244342 | 243809 | 534 | 177 | AUG | UAA |
| ORF384 | – | 245153 | 244383 | 771 | 256 | AUG | UAA |
| ORF385 | – | 245844 | 245161 | 684 | 227 | GUG | UGA |
| ORF386 | – | 246362 | 245934 | 429 | 142 | AUG | UAG |
| ORF387 | – | 246620 | 246372 | 249 | 82 | AUG | UAA |
| ORF388 | – | 247033 | 246704 | 330 | 109 | AUG | UAA |
| ORF389 | – | 247500 | 247030 | 471 | 156 | AUG | UGA |
| ORF390 | – | 247961 | 247497 | 465 | 154 | AUG | UGA |
| Notes: bp = base pair, aa = amino acid | | | | | | | |

Table S2: Putative CDS function of Bacteriophage ValKK3

BLASTn PSI-BLAST Description

ORF Name Homology Score E-Value Identity (%) Homology Score E-Value Identity (%) (With Additional information from Pfam, ScanProsite, NCBI CDD, Interpro)

| ORF1 | Vibrio phage VH7D, complete genome | 521 | 2.00E-144 | 94% | hypothetical protein [Vibrio phage VH7D] | 231 | 1.00E-75 | 97% | – |
| --- | --- | --- | --- | --- | --- | --- | --- | --- | --- |
| ORF2 | Vibrio phage VH7D, complete genome | 538 | 2.00E-149 | 96% | hypothetical protein [Vibrio phage VH7D] | 223 | 2.00E-72 | 97% | Aspartate aminotransferase (AAT) |
| ORF3 | Vibrio phage VH7D, complete genome | 521 | 2.00E-144 | 93% | hypothetical protein [Vibrio phage VH7D] | 230 | 2.00E-75 | 95% | – |
| ORF4 | Vibrio phage VH7D, complete genome | 449 | 7.00E-123 | 99% | hypothetical protein [Vibrio phage VH7D] | 164 | 3.00E-50 | 98% | – |
| ORF5 | Vibrio phage VH7D, complete genome | 920 | 0 | 98% | hypothetical protein [Vibrio phage VH7D] | 364 | 3.00E-126 | 98% | – |
| ORF6 | Vibrio phage VH7D, complete genome | 893 | 0 | 100% | hypothetical protein [Vibrio phage VH7D] | 335 | 2.00E-115 | 100% | – |
| ORF7 | Vibrio phage VH7D, complete genome | 1463 | 0 | 99% | adenylate cyclase [Paenibacillus sp. FSL R5-0912] | 57.8 | 4.00E-07 | 27% | Adenylate cyclase |
| ORF8 | Vibrio phage VH7D, complete genome | 527 | 3.00E-146 | 99% | hypothetical protein [Vibrio phage VH7D] | 194 | 1.00E-61 | 100% | – |
| ORF9 | Vibrio phage VH7D, complete genome | 477 | 3.00E-131 | 100% | hypothetical protein [Vibrio phage VH7D] | 119 | 6.00E-33 | 100% | – |
| ORF10 | Vibrio phage VH7D, complete genome | 826 | 0 | 99% | hypothetical protein [Vibrio phage VH7D] | 309 | 2.00E-105 | 99% | – |
| ORF11 | Vibrio phage VH7D, complete genome | 566 | 8.00E-158 | 99% | hypothetical protein [Vibrio phage VH7D] | 205 | 1.00E-65 | 100% | – |
| ORF12 | Vibrio phage VH7D, complete genome | 316 | 5.00E-83 | 98% | hypothetical protein [Vibrio phage VH7D] | 120 | 6.00E-34 | 98% | – |
| ORF13 | Vibrio phage VH7D, complete genome | 516 | 7.00E-143 | 100% | hypothetical protein [Vibrio phage VH7D] | 190 | 2.00E-60 | 100% | – |
| ORF14 | Vibrio phage VH7D, complete genome | 571 | 2.00E-159 | 95% | hypothetical protein [Vibrio phage VH7D] | 240 | 3.00E-79 | 96% | – |
| ORF15 | Vibrio phage VH7D, complete genome | 665 | 0 | 99% | hypothetical protein [Vibrio phage VH7D] | 253 | 3.00E-84 | 99% | – |
| ORF16 | Vibrio phage VH7D, complete genome | 571 | 2.00E-159 | 99% | hypothetical protein [Vibrio phage VH7D] | 216 | 5.00E-70 | 98% | – |
| ORF17 | Vibrio phage VH7D, complete genome | 1214 | 0 | 96% | hypothetical protein [Vibrio phage VH7D] | 499 | 2.00E-177 | 96% | – |
| ORF18 | Vibrio phage VH7D, complete genome | 255 | 1.00E-64 | 94% | hypothetical protein [Vibrio phage VH7D] | 103 | 3.00E-27 | 89% | – |
| ORF19 | Vibrio phage VH7D, complete genome | 477 | 3.00E-131 | 97% | hypothetical protein [Vibrio phage VH7D] | 186 | 1.00E-58 | 96% | Sugar transport proteins signature 2 |
| ORF20 | Vibrio phage VH7D, complete genome | 388 | 1.00E-104 | 96% | hypothetical protein [Vibrio phage VH7D] | 155 | 8.00E-47 | 100% | – |
| ORF21 | Vibrio phage VH7D, complete genome | 165 | 2.00E-37 | 80% | hypothetical protein [Vibrio phage VH7D] | 144 | 7.00E-43 | 92% | – |
| ORF22 | Vibrio phage VH7D, complete genome | 577 | 4.00E-161 | 95% | hypothetical protein [Vibrio phage VH7D] | 246 | 1.00E-81 | 98% | – |
| ORF23 | Vibrio phage VH7D, complete genome | 394 | 3.00E-106 | 99% | hypothetical protein [Vibrio phage VH7D] | 139 | 7.00E-41 | 99% | – |
| ORF24 | Vibrio phage VH7D, complete genome | 488 | 2.00E-134 | 99% | hypothetical protein [Vibrio phage VH7D] | 185 | 2.00E-58 | 99% | – |
| ORF25 | Vibrio phage VH7D, complete genome | 309 | 9.00E-81 | 96% | hypothetical protein [Vibrio phage VH7D] | 124 | 4.00E-35 | 92% | – |
| ORF26 | Vibrio phage VH7D, complete genome | 848 | 0 | 97% | hypothetical protein [Vibrio phage VH7D] | 331 | 1.00E-113 | 96% | – |
| ORF27 | Vibrio phage VH7D, complete genome | 424 | 5.00E-115 | 93% | hypothetical protein [Vibrio phage VH7D] | 183 | 2.00E-57 | 94% | Uncharacterized ATPase, putative transposase [General function prediction only] |
| ORF28 | Vibrio phage VH7D, complete genome | 676 | 0 | 96% | hypothetical protein [Vibrio phage VH7D] | 279 | 5.00E-94 | 98% | – |
| ORF29 | Vibrio phage VH7D, complete genome | 1031 | 0 | 94% | nicotinamide riboside transporter [Vibrio phage VH7D] | 447 | 1.00E-157 | 97% | Nicotinamide riboside transporter |
| ORF30 | Vibrio phage VH7D, complete genome | 444 | 3.00E-121 | 97% | hypothetical protein [Vibrio phage VH7D] | 169 | 3.00E-52 | 95% | – |
| ORF31 | Vibrio phage VH7D, complete genome | 588 | 2.00E-164 | 97% | hypothetical protein [Vibrio phage VH7D] | 179 | 2.00E-55 | 96% | – |
| ORF32 | Vibrio phage VH7D, complete genome | 544 | 4.00E-151 | 96% | hypothetical protein [Vibrio phage VH7D] | 209 | 2.00E-67 | 96% | – |
| ORF33 | Vibrio phage VH7D, complete genome | 1415 | 0 | 93% | trifunctional NAD biosynthesis/regulator protein NadR [Vibrio phage VH7D] | 667 | 0 | 98% | Trifunctional NAD biosynthesis/regulator protein NadR |
| ORF34 | No Significant Homology | – | – | – | hypothetical protein [Vibrio phage VH7D] | 74.7 | 3.00E-15 | 63% | – |
| ORF35 | No Significant Homology | – | – | – | hypothetical protein [Vibrio phage VH7D] | 127 | 6.00E-36 | 85% | – |
| ORF36 | Vibrio phage VH7D, complete genome | 848 | 0 | 99% | hypothetical protein [Vibrio phage VH7D] | 310 | 9.00E-106 | 98% | – |
| ORF37 | Vibrio phage VH7D, complete genome | 355 | 1.00E-94 | 95% | hypothetical protein [Vibrio phage VH7D] | 157 | 9.00E-48 | 100% | – |
| ORF38 | Vibrio phage VH7D, complete genome | 556 | 6.00E-155 | 95% | hypothetical protein KVP40.0207 [Vibrio phage KVP40] | 175 | 1.00E-53 | 70% | – |
| ORF39 | Vibrio phage VH7D, complete genome | 765 | 0 | 98% | hypothetical protein [Vibrio phage VH7D] | 297 | 7.00E-101 | 99% | – |
| ORF40 | Vibrio phage VH7D, complete genome | 704 | 0 | 97% | DenV Endonuclease V [Vibrio phage KVP40] | 278 | 2.00E-93 | 95% | DenV Endonuclease V |
| ORF41 | Vibrio phage VH7D, complete genome | 427 | 3.00E-116 | 95% | hypothetical protein [Vibrio phage VH7D] | 176 | 9.00E-55 | 93% | – |
| ORF42 | Vibrio phage VH7D, complete genome | 361 | 4.00E-96 | 90% | hypothetical protein [Vibrio phage VH7D] | 176 | 1.00E-54 | 90% | – |
| ORF43 | Vibrio phage VH7D, complete genome | 510 | 4.00E-141 | 98% | hypothetical protein [Vibrio phage VH7D] | 199 | 8.00E-64 | 99% | – |
| ORF44 | Vibrio phage VH7D, complete genome | 1064 | 0 | 96% | hypothetical protein [Vibrio phage VH7D] | 445 | 8.00E-157 | 98% | – |
| ORF45 | Vibrio phage VH7D, complete genome | 649 | 0 | 96% | hypothetical protein [Vibrio phage VH7D] | 248 | 7.00E-82 | 94% | – |
| ORF46 | Vibrio phage VH7D, complete genome | 915 | 0 | 94% | hypothetical protein [Vibrio phage VH7D] | 409 | 2.00E-143 | 97% | – |
| ORF47 | Vibrio phage VH7D, complete genome | 621 | 2.00E-174 | 95% | hypothetical protein [Vibrio phage VH7D] | 216 | 1.00E-69 | 95% | – |
| ORF48 | Vibrio phage VH7D, complete genome | 154 | 1.00E-33 | 79% | hypothetical protein [Vibrio phage VH7D] | 239 | 7.00E-77 | 62% | – |
| ORF49 | No Significant Homology | – | – | – | hypothetical protein [Vibrio phage VH7D] | 67 | 1.00E-10 | 27% | – |
| ORF50 | Vibrio phage VH7D, complete genome | 311 | 2.00E-81 | 99% | hypothetical protein [Vibrio phage VH7D] | 112 | 5.00E-31 | 98% | – |
| ORF51 | Vibrio phage VH7D, complete genome | 438 | 1.00E-119 | 99% | hypothetical protein [Vibrio phage VH7D] | 154 | 2.00E-46 | 96% | – |
| ORF52 | Vibrio phage VH7D, complete genome | 466 | 7.00E-128 | 99% | hypothetical protein [Vibrio phage VH7D] | 179 | 2.00E-56 | 100% | – |
| ORF53 | Vibrio phage VH7D, complete genome | 416 | 7.00E-113 | 96% | hypothetical protein [Vibrio phage VH7D] | 174 | 4.00E-54 | 99% | – |
| ORF54 | Vibrio phage VH7D, complete genome | 532 | 8.00E-148 | 98% | hypothetical protein [Vibrio phage VH7D] | 206 | 1.00E-66 | 98% | – |
| ORF55 | Vibrio phage VH7D, complete genome | 821 | 0 | 92% | hypothetical protein [Vibrio phage VH7D] | 360 | 5.00E-124 | 91% | – |
| ORF56 | Vibrio phage VH7D, complete genome | 538 | 2.00E-149 | 99% | hypothetical protein [Vibrio phage VH7D] | 196 | 1.00E-62 | 100% | – |
| ORF57 | Vibrio phage VH7D, complete genome | 521 | 2.00E-144 | 99% | hypothetical protein [Vibrio phage VH7D] | 183 | 1.00E-57 | 96% | – |
| ORF58 | Vibrio phage VH7D, complete genome | 527 | 4.00E-146 | 96% | hypothetical protein [Vibrio phage VH7D] | 220 | 1.00E-71 | 97% | – |
| ORF59 | Vibrio phage VH7D, complete genome | 643 | 0 | 100% | hypothetical protein [Vibrio phage VH7D] | 236 | 1.00E-77 | 100% | – |
| ORF60 | Vibrio phage VH7D, complete genome | 560 | 4.00E-156 | 100% | hypothetical protein [Vibrio phage VH7D] | 211 | 2.00E-68 | 100% | – |
| ORF61 | Vibrio phage VH7D, complete genome | 998 | 0 | 98% | thymidine kinase [Vibrio phage VH7D] | 395 | 5.00E-138 | 99% | Thymidine kinase |
| ORF62 | Vibrio phage VH7D, complete genome | 555 | 2.00E-154 | 97% | hypothetical protein [Vibrio phage VH7D] | 214 | 3.00E-69 | 94% | – |
| ORF63 | Vibrio phage VH7D, complete genome | 1613 | 0 | 98% | hypothetical protein [Vibrio phage VH7D] | 639 | 0 | 99% | N-terminal glutamine amidase |
| ORF64 | Vibrio phage VH7D, complete genome | 588 | 2.00E-164 | 99% | hypothetical protein [Vibrio phage VH7D] | 213 | 7.00E-69 | 100% | – |
| ORF65 | Vibrio phage VH7D, complete genome | 267 | 4.00E-68 | 97% | hypothetical protein [Vibrio phage VH7D] | 107 | 4.00E-29 | 98% | – |

BLASTn PSI-BLAST Description

ORF Name Homology Score E-Value Identity (%) Homology Score E-Value Identity (%) (With Additional information from Pfam, ScanProsite, NCBI CDD, Interpro)

| ORF66 | Vibrio phage VH7D, complete genome | 289 | 1.00E-74 | 95% | hypothetical protein [Vibrio phage VH7D] | 124 | 2.00E-35 | 97% | – |
| --- | --- | --- | --- | --- | --- | --- | --- | --- | --- |
| ORF67 | Vibrio phage VH7D, complete genome | 749 | 0 | 98% | hypothetical protein [Vibrio phage VH7D] | 294 | 1.00E-99 | 97% | – |
| ORF68 | Vibrio phage VH7D, complete genome | 289 | 9.00E-75 | 100% | hypothetical protein VPFG_00244 [Vibrio phage nt-1] | 91.3 | 1.00E-22 | 82% | – |
| ORF69 | Vibrio phage VH7D, complete genome | 372 | 1.00E-99 | 99% | hypothetical protein [Vibrio phage VH7D] | 136 | 1.00E-39 | 100% | – |
| ORF70 | Vibrio phage VH7D, complete genome | 560 | 4.00E-156 | 97% | hypothetical protein [Vibrio phage VH7D] | 215 | 1.00E-69 | 97% | – |
| ORF71 | Vibrio phage VH7D, complete genome | 344 | 3.00E-91 | 95% | hypothetical protein KVP40.0183 [Vibrio phage KVP40] | 79.7 | 2.00E-17 | 66% | – |
| ORF72 | No Significant Homology | – | – | – | hypothetical protein pp2_179 [Vibriophage phi-pp2] | 91.7 | 2.00E-21 | 45% | – |
| ORF73 | Vibrio phage VH7D, complete genome | 405 | 1.00E-109 | 97% | hypothetical protein [Vibrio phage VH7D] | 155 | 5.00E-47 | 94% | – |
| ORF74 | Vibriophage phi-pp2, complete genome | 169 | 2.00E-38 | 91% | hypothetical protein pp2_178 [Vibriophage phi-pp2] | 96.7 | 6.00E-24 | 73% | – |
| ORF75 | Vibrio phage VH7D, complete genome | 575 | 2.00E-160 | 96% | hypothetical protein [Vibrio phage VH7D] | 237 | 6.00E-78 | 95% | – |
| ORF76 | No Significant Homology | – | – | – | hypothetical protein VPFG_00231 [Vibrio phage nt-1] | 49.7 | 2.00E-05 | 36% | – |
| ORF77 | Vibrio phage VH7D, complete genome | 582 | 1.00E-162 | 93% | hypothetical protein [Vibrio phage VH7D] | 268 | 1.00E-89 | 93% | – |
| ORF78 | Vibrio phage VH7D, complete genome | 449 | 1.00E-122 | 88% | hypothetical protein [Vibrio phage VH7D] | 218 | 4.00E-70 | 86% | – |
| ORF79 | Vibrio phage VH7D, complete genome | 798 | 0 | 99% | hypothetical protein [Vibrio phage VH7D] | 298 | 2.00E-101 | 100% | – |
| ORF80 | Vibrio phage VH7D, complete genome | 460 | 3.00E-126 | 99% | hypothetical protein [Vibrio phage VH7D] | 168 | 6.00E-52 | 99% | – |
| ORF81 | Vibrio phage VH7D, complete genome | 311 | 2.00E-81 | 99% | hypothetical protein [Vibrio phage VH7D] | 112 | 1.00E-30 | 96% | – |
| ORF82 | Vibrio phage VH7D, complete genome | 721 | 0 | 99% | hypothetical protein [Vibrio phage VH7D] | 272 | 3.00E-91 | 99% | – |
| ORF83 | Vibrio phage VH7D, complete genome | 521 | 2.00E-144 | 96% | hypothetical protein [Vibrio phage VH7D] | 211 | 3.00E-68 | 96% | – |
| ORF84 | Vibrio phage VH7D, complete genome | 1110 | 0 | 99% | hypothetical protein [Vibrio phage VH7D] | 417 | 4.00E-146 | 100% | – |
| ORF85 | Vibrio phage VH7D, complete genome | 555 | 2.00E-154 | 99% | hypothetical protein [Vibrio phage VH7D] | 213 | 5.00E-69 | 100% | – |
| ORF86 | Vibrio phage VH7D, complete genome | 582 | 8.00E-163 | 99% | hypothetical protein [Vibrio phage VH7D] | 218 | 5.00E-71 | 99% | – |
| ORF87 | Vibrio phage VH7D, complete genome | 215 | 2.00E-52 | 92% | hypothetical protein KVP40.0172 [Vibrio phage KVP40] | 80.1 | 3.00E-18 | 73% | – |
| ORF88 | Vibrio phage VH7D, complete genome | 322 | 1.00E-84 | 95% | hypothetical protein [Vibrio phage VH7D] | 143 | 1.00E-42 | 94% | – |
| ORF89 | Vibrio phage VH7D, complete genome | 272 | 9.00E-70 | 96% | No Significant Homology | – | – | – | – |
| ORF90 | Vibrio phage VH7D, complete genome | 100 | 5.00E-18 | 85% | No Significant Homology | – | – | – | – |
| ORF91 | No Significant Homology | – | – | – | No Significant Homology | – | – | – | – |
| ORF92 | Vibriophage phi-pp2, complete genome | 276 | 2.00E-70 | 85% | hypothetical protein pp2_165 [Vibriophage phi-pp2] | 179 | 1.00E-55 | 77% | – |
| ORF93 | No Significant Homology | – | – | – | hypothetical protein [Vibrio phage VH7D] | 106 | 2.00E-28 | 89% | – |
| ORF94 | Vibrio phage VH7D, complete genome | 344 | 3.00E-91 | 96% | hypothetical protein [Vibrio phage VH7D] | 138 | 2.00E-40 | 97% | – |
| ORF95 | Vibrio phage VH7D, complete genome | 339 | 1.00E-89 | 95% | hypothetical protein [Vibrio phage VH7D] | 142 | 3.00E-42 | 97% | – |
| ORF96 | Vibrio phage VH7D, complete genome | 339 | 1.00E-89 | 97% | hypothetical protein KVP40.0165 [Vibrio phage KVP40] | 113 | 9.00E-31 | 82% | – |
| ORF97 | Vibrio phage VH7D, complete genome | 1070 | 0 | 97% | nicotinate-nucleotide adenylyltransferase [Desulfococcus multivorans] | 79.7 | 6.00E-14 | 38% | Nicotinate-nucleotide adenylyltransferase |
| ORF98 | Vibrio phage VH7D, complete genome | 616 | 9.00E-173 | 98% | hypothetical protein [Vibrio phage VH7D] | 238 | 3.00E-78 | 97% | – |
| ORF99 | Vibrio phage VH7D, complete genome | 505 | 2.00E-139 | 100% | hypothetical protein [Vibrio phage VH7D] | 185 | 3.00E-58 | 100% | – |
| ORF100 | Vibrio phage VH7D, complete genome | 1801 | 0 | 98% | bifunctional NMN adenylyltransferase/nudix hydrolase [Vibrio phage VH7D] | 714 | 0 | 99% | Bifunctional NMN adenylyltransferase/nudix hydrolase |
| ORF101 | Vibrio phage VH7D, complete genome | 881 | 0 | 97% | hypothetical protein [Vibrio phage VH7D] | 346 | 3.00E-119 | 98% | – |
| ORF102 | Vibrio phage VH7D, complete genome | 854 | 0 | 100% | hypothetical protein [Vibrio phage VH7D] | 300 | 1.00E-101 | 100% | – |
| ORF103 | Vibrio phage VH7D, complete genome | 1391 | 0 | 99% | hypothetical protein [Vibrio phage VH7D] | 514 | 0 | 99% | – |
| ORF104 | Vibrio phage VH7D, complete genome | 327 | 2.00E-86 | 99% | hypothetical protein [Vibrio phage VH7D] | 122 | 1.00E-34 | 98% | – |
| ORF105 | Vibrio phage VH7D, complete genome | 560 | 4.00E-156 | 99% | hypothetical protein [Vibrio phage VH7D] | 211 | 5.00E-68 | 97% | – |
| ORF106 | Vibrio phage VH7D, complete genome | 2333 | 0 | 99% | ATP-dependent DNA helicase [Vibrio phage VH7D] | 881 | 0 | 99% | ATP-dependent DNA helicase |
| ORF107 | Vibrio phage VH7D, complete genome | 693 | 0 | 100% | hypothetical protein pp2_150 [Vibriophage phi-pp2] | 246 | 2.00E-81 | 94% | Protein of unknown function (DUF2493) |
| ORF108 | Vibrio phage VH7D, complete genome | 837 | 0 | 99% | hypothetical protein [Vibrio phage VH7D] | 290 | 5.00E-98 | 100% | – |
| ORF109 | Vibrio phage VH7D, complete genome | 460 | 3.00E-126 | 99% | hypothetical protein [Vibrio phage VH7D] | 169 | 2.00E-52 | 99% | – |
| ORF110 | Vibrio phage VH7D, complete genome | 1873 | 0 | 100% | putative 38.9 kDa protein [Vibrio phage VH7D] | 706 | 0.00E+00 | 100% | – |
| ORF111 | Vibrio phage VH7D, complete genome | 510 | 3.00E-141 | 100% | hypothetical protein KVP40.0152 [Vibrio phage KVP40] | 176 | 5.00E-55 | 91% | – |
| ORF112 | Vibrio phage VH7D, complete genome | 1026 | 0 | 99% | hypothetical protein [Vibrio phage VH7D] | 217 | 6.00E-68 | 62% | – |
| ORF113 | Vibrio phage VH7D, complete genome | 909 | 0 | 100% | hypothetical protein [Vibrio phage VH7D] | 320 | 4.00E-109 | 100% | – |
| ORF114 | Vibrio phage VH7D, complete genome | 1186 | 0 | 98% | potassium voltage-gated channel subfamily A member 1 protein [Vibrio phage VH7D] | 419 | 2.00E-146 | 98% | Potassium voltage-gated channel subfamily A member 1 protein |
| ORF115 | Vibrio phage VH7D, complete genome | 638 | 2.00E-179 | 100% | hypothetical protein [Vibrio phage VH7D] | 234 | 5.00E-77 | 100% | – |
| ORF116 | Vibrio phage VH7D, complete genome | 460 | 3.00E-126 | 99% | hypothetical protein [Vibrio phage VH7D] | 172 | 2.00E-53 | 99% | – |
| ORF117 | Vibrio phage VH7D, complete genome | 1227 | 0 | 88% | protein rIIB [Vibrio phage VH7D] | 656 | 0 | 92% | Protein rIIB |
| ORF118 | Vibrio phage VH7D, complete genome | 2687 | 0 | 90% | protein rIIA [Vibrio phage VH7D] | 1346 | 0 | 94% | Protein rIIA |
| ORF119 | Vibrio phage VH7D, complete genome | 255 | 8.00E-65 | 100% | hypothetical protein [Vibrio phage VH7D] | 91.3 | 9.00E-23 | 100% | – |
| ORF120 | Vibrio phage VH7D, complete genome | 503 | 6.00E-139 | 97% | hypothetical protein [Vibrio phage VH7D] | 194 | 1.00E-61 | 97% | – |
| ORF121 | Vibrio phage VH7D, complete genome | 494 | 3.00E-136 | 99% | hypothetical protein [Vibrio phage VH7D] | 187 | 2.00E-59 | 99% | – |
| ORF122 | Vibrio phage VH7D, complete genome | 643 | 0 | 99% | hypothetical protein [Vibrio phage VH7D] | 230 | 4.00E-75 | 100% | Domain of unknown function (DUF4098) |
| ORF123 | Vibrio phage VH7D, complete genome | 399 | 6.00E-108 | 100% | hypothetical protein [Vibrio phage VH7D] | 146 | 8.00E-44 | 100% | – |
| ORF124 | Vibrio phage VH7D, complete genome | 881 | 0 | 94% | DNA methyltransferase [Vibrio phage nt-1] | 335 | 4.00E-114 | 82% | DNA methyltransferase |
| ORF125 | Vibrio phage VH7D, complete genome | 845 | 0 | 96% | hypothetical protein [Vibrio phage VH7D] | 336 | 3.00E-115 | 93% | – |
| ORF126 | Vibrio phage VH7D, complete genome | 1009 | 0 | 99% | hypothetical protein [Vibrio phage VH7D] | 381 | 9.00E-133 | 100% | – |
| ORF127 | Vibrio phage VH7D, complete genome | 1840 | 0 | 99% | RNA ligase 2 [Vibrio phage VH7D] | 687 | 0 | 99% | RNA ligase 2 |
| ORF128 | Vibrio phage VH7D, complete genome | 453 | 5.00E-124 | 99% | hypothetical protein [Vibrio phage VH7D] | 160 | 1.00E-48 | 98% | – |
| ORF129 | Vibrio phage VH7D, complete genome | 926 | 0 | 99% | recombination endonuclease VII [Vibriophage phi-pp2] | 333 | 2.00E-114 | 93% | T4 Recombination endonuclease VII |
| ORF130 | Vibrio phage VH7D, complete genome | 715 | 0 | 99% | hypothetical protein [Vibrio phage VH7D] | 261 | 3.00E-87 | 100% | – |

BLASTn PSI-BLAST Description

ORF Name Homology Score E-Value Identity (%) Homology Score E-Value Identity (%) (With Additional information from Pfam, ScanProsite, NCBI CDD, Interpro)

| ORF131 | Vibrio phage VH7D, complete genome | 627 | 4.00E-176 | 100% | head assembly cochaperone with GroE [Vibrio phage KVP40] | 209 | 4.00E-67 | 95% | Head assembly cochaperone |
| --- | --- | --- | --- | --- | --- | --- | --- | --- | --- |
| ORF132 | Vibrio phage VH7D, complete genome | 383 | 5.00E-103 | 100% | hypothetical protein [Vibrio phage VH7D] | 138 | 1.00E-40 | 100% | Aerobic respiration control sensor protein ArcB |
| ORF133 | Vibrio phage VH7D, complete genome | 366 | 6.00E-98 | 98% | hypothetical protein [Vibrio phage VH7D] | 136 | 8.00E-40 | 93% | – |
| ORF134 | Vibrio phage VH7D, complete genome | 483 | 7.00E-133 | 98% | hypothetical protein [Vibrio phage VH7D] | 187 | 4.00E-59 | 100% | Protein of unknown function (viral family of protein) |
| ORF135 | Vibrio phage VH7D, complete genome | 1103 | 0 | 99% | hypothetical protein [Vibrio phage VH7D] | 424 | 6.00E-149 | 99% | – |
| ORF136 | Vibrio phage VH7D, complete genome | 1293 | 0 | 99% | 7-cyano-7-deazaguanine synthase [Vibrio phage VH7D] | 494 | 1.00E-175 | 99% | 7-cyano-7-deazaguanine synthase |
| ORF137 | Vibrio phage VH7D, complete genome | 1591 | 0 | 98% | NADPH-dependent 7-cyano-7-deazaguanine reductase [Vibrio phage VH7D] | 629 | 0 | 99% | NADPH-dependent 7-cyano-7-deazaguanine reductase |
| ORF138 | Vibrio phage VH7D, complete genome | 2322 | 0 | 97% | hypothetical protein [Vibrio phage VH7D] | 930 | 0 | 98% | – |
| ORF139 | Vibrio phage VH7D, complete genome | 1230 | 0 | 99% | GTP cyclohydrolase 1 [Vibrio phage VH7D] | 463 | 7.00E-164 | 100% | GTP cyclohydrolase 1 |
| ORF140 | Vibrio phage VH7D, complete genome | 1685 | 0 | 99% | 6-pyruvoyl tetrahydropterin synthase-like protein [Vibrio phage nt-1] | 483 | 1.00E-168 | 76% | 6-pyruvoyl tetrahydropterin synthase-like protein |
| ORF141 | Vibrio phage VH7D, complete genome | 682 | 0 | 94% | deoxycytidylate deaminase [Vibrio phage VH7D] | 294 | 2.00E-99 | 91% | Deoxycytidylate deaminase |
| ORF142 | Vibrio phage VH7D, complete genome | 566 | 8.00E-158 | 99% | hypothetical protein [Vibrio phage VH7D] | 211 | 3.00E-68 | 98% | – |
| ORF143 | Vibrio phage VH7D, complete genome | 544 | 4.00E-151 | 99% | hypothetical protein [Vibrio phage VH7D] | 203 | 3.00E-65 | 99% | – |
| ORF144 | Vibrio phage VH7D, complete genome | 361 | 2.00E-96 | 100% | hypothetical protein [Vibrio phage VH7D] | 133 | 1.00E-38 | 100% | – |
| ORF145 | Vibrio phage VH7D, complete genome | 1563 | 0 | 99% | hypothetical protein [Vibrio phage VH7D] | 593 | 0 | 99% | – |
| ORF146 | Vibrio phage VH7D, complete genome | 1594 | 0 | 98% | hypothetical protein [Vibrio phage VH7D] | 621 | 0 | 99% | – |
| ORF147 | Vibrio phage VH7D, complete genome | 135 | 4.00E-28 | 90% | hypothetical protein [Vibrio phage VH7D] | 173 | 3.00E-50 | 57% | – |
| ORF148 | Vibrio phage VH7D, complete genome | 510 | 4.00E-141 | 98% | hypothetical protein [Vibrio phage VH7D] | 198 | 3.00E-63 | 100% | – |
| ORF149 | Vibrio phage VH7D, complete genome | 510 | 3.00E-141 | 99% | conserved hypothetical protein [Vibrio phage KVP40] | 196 | 9.00E-63 | 99% | Domain of unknown function (DUF4326) |
| ORF150 | Vibrio phage VH7D, complete genome | 566 | 8.00E-158 | 100% | hypothetical protein [Vibrio phage VH7D] | 205 | 6.00E-66 | 100% | – |
| ORF151 | Vibrio phage VH7D, complete genome | 185 | 1.00E-43 | 100% | hypothetical protein KVP40.0111 [Vibrio phage KVP40] | 80.5 | 3.00E-18 | 64% | – |
| ORF152 | Vibrio phage VH7D, complete genome | 2239 | 0 | 96% | hypothetical protein [Vibrio phage VH7D] | 949 | 0 | 98% | – |
| ORF153 | Vibrio phage VH7D, complete genome | 521 | 2.00E-144 | 100% | hypothetical protein [Vibrio phage VH7D] | 190 | 3.00E-60 | 100% | – |
| ORF154 | Vibrio phage VH7D, complete genome | 621 | 2.00E-174 | 100% | hypothetical protein [Vibrio phage VH7D] | 230 | 1.00E-75 | 100% | – |
| ORF155 | Vibrio phage VH7D, complete genome | 416 | 6.00E-113 | 98% | hypothetical protein [Vibrio phage VH7D] | 101 | 7.00E-26 | 62% | – |
| ORF156 | Vibrio phage VH7D, complete genome | 643 | 0 | 96% | hypothetical protein [Vibrio phage VH7D] | 262 | 1.00E-87 | 96% | – |
| ORF157 | Vibrio phage VH7D, complete genome | 1253 | 0 | 99% | hypothetical protein [Vibrio phage VH7D] | 472 | 5.00E-167 | 99% | – |
| ORF158 | Vibrio phage VH7D, complete genome | 1469 | 0 | 92% | hypothetical protein VPFG_00150 [Vibrio phage nt-1] | 649 | 0 | 87% | – |
| ORF159 | Vibrio phage VH7D, complete genome | 750 | 0 | 89% | hypothetical protein [Vibrio phage VH7D] | 382 | 2.00E-132 | 88% | – |
| ORF160 | Vibrio phage VH7D, complete genome | 623 | 6.00E-175 | 97% | hypothetical protein pp2_098 [Vibriophage phi-pp2] | 259 | 3.00E-86 | 97% | – |
| ORF161 | Vibrio phage VH7D, complete genome | 1070 | 0 | 98% | hypothetical protein [Vibrio phage VH7D] | 425 | 2.00E-149 | 99% | – |
| ORF162 | Vibrio phage VH7D, complete genome | 350 | 8.00E-93 | 90% | hypothetical protein [Vibrio phage VH7D] | 162 | 2.00E-49 | 91% | – |
| ORF163 | Vibrio phage VH7D, complete genome | 1873 | 0 | 90% | hypothetical protein [Vibrio phage VH7D] | 910 | 0 | 92% | – |
| ORF164 | Vibrio phage VH7D, complete genome | 737 | 0 | 100% | hypothetical protein [Vibrio phage VH7D] | 264 | 4.00E-88 | 100% | – |
| ORF165 | Vibrio phage VH7D, complete genome | 355 | 1.00E-94 | 100% | hypothetical protein [Vibrio phage VH7D] | 131 | 5.00E-38 | 100% | – |
| ORF166 | Vibrio phage VH7D, complete genome | 427 | 3.00E-116 | 100% | hypothetical protein [Vibrio phage VH7D] | 157 | 5.00E-48 | 100% | – |
| ORF167 | Vibrio phage VH7D, complete genome | 267 | 5.00E-68 | 95% | hypothetical protein KVP40.0093 [Vibrio phage KVP40] | 83.2 | 2.00E-19 | 73% | – |
| ORF168 | Vibrio phage VH7D, complete genome | 604 | 3.00E-169 | 88% | putative 17.5 kDa protein [Vibrio phage VH7D] | 328 | 4.00E-112 | 91% | Protein with Macro domain |
| ORF169 | Vibrio phage VH7D, complete genome | 946 | 0 | 99% | hypothetical protein [Vibrio phage VH7D] | 350 | 1.00E-120 | 97% | – |
| ORF170 | Vibrio phage VH7D, complete genome | 1613 | 0 | 98% | polynucleotide kinase [Vibrio phage VH7D] | 626 | 0 | 99% | Polynucleotide kinase |
| ORF171 | Vibrio phage VH7D, complete genome | 189 | 1.00E-44 | 81% | hypothetical protein VPFG_00137 [Vibrio phage nt-1] | 130 | 2.00E-37 | 84% | – |
| ORF172 | Vibrio phage VH7D, complete genome | 529 | 1.00E-146 | 99% | hypothetical protein [Vibrio phage VH7D] | 195 | 5.00E-62 | 99% | – |
| ORF173 | Vibrio phage VH7D, complete genome | 604 | 2.00E-169 | 99% | hypothetical protein [Vibrio phage VH7D] | 202 | 2.00E-64 | 100% | – |
| ORF174 | Vibrio phage VH7D, complete genome | 649 | 0 | 99% | hypothetical protein [Vibrio phage VH7D] | 234 | 5.00E-77 | 99% | – |
| ORF175 | Vibrio phage VH7D, complete genome | 499 | 7.00E-138 | 100% | hypothetical protein [Vibrio phage VH7D] | 181 | 5.00E-57 | 100% | – |
| ORF176 | Vibrio phage VH7D, complete genome | 2095 | 0 | 99% | RNA ligase [Vibrio phage VH7D] | 794 | 0 | 99% | RNA ligase |
| ORF177 | Vibrio phage VH7D, complete genome | 488 | 2.00E-134 | 99% | hypothetical protein [Vibrio phage VH7D] | 176 | 7.00E-55 | 99% | – |
| ORF178 | Vibrio phage VH7D, complete genome | 4671 | 0 | 99% | DNA polymerase [Vibrio phage VH7D] | 1762 | 0 | 100% | DNA polymerase |
| ORF179 | No Significant Homology | – | – | – | No Significant Homology | – | – | – | – |
| ORF180 | Vibrio phage VH7D, complete genome | 508 | 2.00E-140 | 92% | RegA [Vibrio phage KVP40] | 258 | 4.00E-86 | 99% | RegA |
| ORF181 | Vibrio phage VH7D, complete genome | 904 | 0 | 99% | DNA polymerase accessory protein 62 [Vibrio phage VH7D] | 336 | 1.00E-115 | 100% | DNA polymerase accessory protein |
| ORF182 | Vibrio phage VH7D, complete genome | 1735 | 0 | 99% | DNA polymerase accessory protein 44 [Vibrio phage VH7D] | 653 | 0 | 100% | DNA polymerase accessory protein |
| ORF183 | Vibrio phage VH7D, complete genome | 1219 | 0 | 99% | DNA polymerase processivity component [Vibrio phage VH7D] | 448 | 7.00E-158 | 100% | DNA polymerase processivity component |
| ORF184 | Vibrio phage VH7D, complete genome | 388 | 1.00E-104 | 100% | hypothetical protein [Vibrio phage VH7D] | 145 | 4.00E-43 | 100% | – |
| ORF185 | Vibrio phage VH7D, complete genome | 826 | 0 | 99% | hypothetical protein [Vibrio phage VH7D] | 318 | 8.00E-109 | 99% | – |
| ORF186 | Vibrio phage VH7D, complete genome | 3408 | 0 | 94% | exonuclease subunit 2 [Vibrio phage VH7D] | 1466 | 0 | 97% | Exonuclease subunit 2 |
| ORF187 | No Significant Homology | – | – | – | No Significant Homology | – | – | – | – |
| ORF188 | No Significant Homology | – | – | – | hypothetical protein VPFG_00120 [Vibrio phage nt-1] | 154 | 8.00E-47 | 95% | – |
| ORF189 | Vibrio phage VH7D, complete genome | 1103 | 0 | 86% | exonuclease subunit 1 [Vibrio phage VH7D] | 694 | 0 | 95% | Exonuclease subunit 1 |
| ORF190 | Vibrio phage VH7D, complete genome | 617 | 4.00E-173 | 89% | putative 5'(3')-deoxyribonucleotidase [Vibrio phage VH7D] | 328 | 2.00E-112 | 92% | Putative 5'(3')-deoxyribonucleotidase |
| ORF191 | Vibrio phage VH7D, complete genome | 837 | 0 | 99% | putative 32.4 kDa protein [Vibrio phage VH7D] | 317 | 3.00E-108 | 98% | Bacteriophage protein GP30.3 NADAR Superfamily |
| ORF192 | Vibrio phage VH7D, complete genome | 643 | 0 | 99% | putative 12.4 kDa protein [Vibrio phage VH7D] | 231 | 9.00E-76 | 100% | Protein of unknown function (DUF2654) |
| ORF193 | Vibrio phage VH7D, complete genome | 854 | 0 | 100% | hypothetical protein [Vibrio phage VH7D] | 310 | 8.00E-106 | 100% | – |
| ORF194 | Vibrio phage VH7D, complete genome | 488 | 2.00E-134 | 99% | hypothetical protein [Vibrio phage VH7D] | 171 | 4.00E-53 | 100% | – |
| ORF195 | Vibrio phage VH7D, complete genome | 1175 | 0 | 99% | hypothetical protein [Vibrio phage VH7D] | 436 | 2.00E-153 | 99% | – |

BLASTn PSI-BLAST Description

ORF Name Homology Score E-Value Identity (%) Homology Score E-Value Identity (%) (With Additional information from Pfam, ScanProsite, NCBI CDD, Interpro)

| ORF196 | Vibrio phage VH7D, complete genome | 2599 | 0 | 99% | hypothetical protein [Vibrio phage VH7D] | 991 | 0 | 99% | – |
| --- | --- | --- | --- | --- | --- | --- | --- | --- | --- |
| ORF197 | Vibrio phage VH7D, complete genome | 909 | 0 | 99% | RNA polymerase sigma factor [Vibrio phage VH7D] | 356 | 3.00E-123 | 99% | RNA polymerase sigma factor |
| ORF198 | Vibrio phage VH7D, complete genome | 1574 | 0 | 98% | head vertex protein [Vibrio phage VH7D] | 608 | 0 | 99% | Head vertex protein |
| ORF199 | Vibrio phage VH7D, complete genome | 444 | 3.00E-121 | 100% | glutaredoxin [Vibrio phage KVP40] | 155 | 8.00E-47 | 90% | Glutaredoxin |
| ORF200 | Vibrio phage VH7D, complete genome | 1469 | 0 | 99% | SprT-like protein [Mycobacterium phage HINdeR] | 80.1 | 5.00E-15 | 47% | SprT-like protein |
| ORF201 | Vibrio phage VH7D, complete genome | 926 | 0 | 99% | hypothetical protein [Vibrio phage VH7D] | 343 | 4.00E-118 | 99% | – |
| ORF202 | Vibrio phage VH7D, complete genome | 2959 | 0 | 99% | RNA polymerase-ADP-ribosyltransferase [Vibriophage phi-pp2] | 818 | 0 | 70% | RNA polymerase-ADP-ribosyltransferase |
| ORF203 | No Significant Homology | – | – | – | hypothetical protein VPFG_00105 [Vibrio phage nt-1] | 260 | 7.00E-82 | 45% | – |
| ORF204 | Vibrio phage VH7D, complete genome | 2422 | 0 | 99% | DNA ligase [Vibrio phage VH7D] | 911 | 0 | 99 | DNA ligase |
| ORF205 | Vibrio phage VH7D, complete genome | 339 | 1.00E-89 | 99% | hypothetical protein [Vibrio phage VH7D] | 128 | 6.00E-37 | 100% | – |
| ORF206 | Vibrio phage VH7D, complete genome | 549 | 8.00E-153 | 99% | hypothetical protein [Vibrio phage VH7D] | 208 | 2.00E-67 | 99% | – |
| ORF207 | Vibrio phage VH7D, complete genome | 1092 | 0 | 99% | hydrolase [Vibrio phage KVP40] | 367 | 2.00E-126 | 89% | Haloacid dehalogenase-like hydrolase |
| ORF208 | Vibrio phage VH7D, complete genome | 361 | 2.00E-96 | 99% | hypothetical protein [Vibrio phage VH7D] | 123 | 9.00E-35 | 98% | – |
| ORF209 | Vibrio phage VH7D, complete genome | 931 | 0 | 99% | hypothetical protein [Vibrio phage VH7D] | 354 | 1.00E-122 | 100% | – |
| ORF210 | Vibrio phage VH7D, complete genome | 455 | 1.00E-124 | 100% | hypothetical protein [Vibrio phage VH7D] | 164 | 2.00E-50 | 100% | – |
| ORF211 | Vibrio phage VH7D, complete genome | 327 | 2.00E-86 | 98% | hypothetical protein [Vibrio phage VH7D] | 102 | 9.00E-27 | 94% | – |
| ORF212 | Vibrio phage VH7D, complete genome | 1325 | 0 | 99% | metallophosphoesterase [Vibrio phage nt-1] | 451 | 2.00E-158 | 86% | Metallophosphoesterase |
| ORF213 | Vibrio phage VH7D, complete genome | 477 | 3.00E-131 | 99 | hypothetical protein [Vibrio phage VH7D] | 183 | 2.00E-57 | 100% | – |
| ORF214 | Vibrio phage VH7D, complete genome | 693 | 0 | 98% | hypothetical protein [Vibrio phage VH7D] | 265 | 8.00E-89 | 98% | – |
| ORF215 | Vibrio phage VH7D, complete genome | 488 | 2.00E-134 | 99% | hypothetical protein VPFG_00095 [Vibrio phage nt-1] | 160 | 1.00E-48 | 93% | – |
| ORF216 | Vibrio phage VH7D, complete genome | 399 | 6.00E-108 | 100% | hypothetical protein [Vibrio phage VH7D] | 142 | 3.00E-42 | 100% | – |
| ORF217 | Vibrio phage VH7D, complete genome | 427 | 3.00E-116 | 100% | hypothetical protein [Vibrio phage VH7D] | 150 | 4.00E-45 | 100% | – |
| ORF218 | Vibrio phage VH7D, complete genome | 3280 | 0 | 99% | DNA topoisomerase large subunit [Vibrio phage VH7D] | 1228 | 0 | 100% | DNA topoisomerase large subunit |
| ORF219 | Vibrio phage VH7D, complete genome | 1336 | 0 | 98% | NAD-dependent deacetylase [Vibrio phage VH7D] | 523 | 0 | 99% | NAD-dependent deacetylase |
| ORF220 | Vibrio phage VH7D, complete genome | 521 | 2.00E-144 | 92% | hypothetical protein [Vibrio phage VH7D] | 234 | 1.00E-76 | 92% | – |
| ORF221 | Vibrio phage VH7D, complete genome | 344 | 3.00E-91 | 90% | hypothetical protein VPFG_00089 [Vibrio phage nt-1] | 158 | 7.00E-48 | 88% | – |
| ORF222 | Vibrio phage VH7D, complete genome | 723 | 0 | 99% | hypothetical protein [Vibrio phage VH7D] | 269 | 4.00E-90 | 100% | – |
| ORF223 | Vibrio phage VH7D, complete genome | 604 | 2.00E-169 | 99% | hypothetical protein [Vibrio phage VH7D] | 225 | 2.00E-73 | 99% | – |
| ORF224 | Vibrio phage VH7D, complete genome | 1286 | 0 | 97% | hypothetical protein [Vibrio phage VH7D] | 523 | 0 | 99% | – |
| ORF225 | Vibrio phage VH7D, complete genome | 322 | 2.00E-84 | 87% | hypothetical protein [Vibrio phage VH7D] | 172 | 3.00E-53 | 88% | – |
| ORF226 | Vibrio phage VH7D, complete genome | 521 | 2.00E-144 | 96% | hypothetical protein KVP40.0035 [Vibrio phage KVP40] | 208 | 6.00E-67 | 97% | – |
| ORF227 | Vibrio phage VH7D, complete genome | 532 | 8.00E-148 | 99% | hypothetical protein [Vibrio phage VH7D] | 199 | 9.00E-64 | 100% | – |
| ORF228 | Vibrio phage VH7D, complete genome | 377 | 3.00E-101 | 99% | hypothetical protein [Vibrio phage VH7D] | 119 | 2.00E-33 | 99% | – |
| ORF229 | Vibrio phage VH7D, complete genome | 355 | 1.00E-94 | 99% | hypothetical protein [Vibrio phage VH7D] | 125 | 1.00E-35 | 98% | – |
| ORF230 | Vibrio phage VH7D, complete genome | 1657 | 0 | 99% | thymidylate synthase [Vibrio phage VH7D] | 620 | 0 | 99% | Thymidylate synthase |
| ORF231 | Vibrio phage VH7D, complete genome | 372 | 1.00E-99 | 99% | hypothetical protein [Vibrio phage VH7D] | 134 | 6.00E-39 | 99% | – |
| ORF232 | Vibrio phage VH7D, complete genome | 422 | 1.00E-114 | 99% | hypothetical protein [Vibrio phage VH7D] | 152 | 5.00E-46 | 99% | – |
| ORF233 | Vibrio phage VH7D, complete genome | 660 | 0 | 94% | hypothetical protein [Vibrio phage VH7D] | 288 | 5.00E-97 | 93% | Peptidyl-tRNA hydrolase |
| ORF234 | No Significant Homology | – | – | – | homing endonuclease [Klebsiella phage KP27] | 140 | 2.00E-37 | 43% | Homing endonuclease |
| ORF235 | Vibrio phage VH7D, complete genome | 453 | 5.00E-124 | 100% | hypothetical protein [Vibrio phage VH7D] | 169 | 3.00E-52 | 100% | – |
| ORF236 | Vibrio phage VH7D, complete genome | 726 | 0.00E+00 | 99% | hypothetical protein [Vibrio phage VH7D] | 261 | 2.00E-87 | 100% | – |
| ORF237 | Vibrio phage VH7D, complete genome | 255 | 8.00E-65 | 100% | hypothetical protein [Vibrio phage VH7D] | 92.4 | 3.00E-23 | 100% | – |
| ORF238 | Vibrio phage VH7D, complete genome | 300 | 4.00E-78 | 100% | hypothetical protein [Vibrio phage VH7D] | 110 | 3.00E-30 | 100% | – |
| ORF239 | Vibrio phage VH7D, complete genome | 1280 | 0 | 100% | hypothetical protein [Vibrio phage VH7D] | 479 | 6.00E-170 | 100% | DnaQ like exonuclease |
| ORF240 | Vibrio phage VH7D, complete genome | 255 | 2.00E-64 | 89% | hypothetical protein [Vibrio phage VH7D] | 119 | 7.00E-33 | 85% | – |
| ORF241 | Vibrio phage VH7D, complete genome | 1081 | 0 | 100% | deoxyuridine 5'-triphosphate nucleotidohydrolase [Vibrio phage VH7D] | 396 | 3.00E-138 | 100% | Deoxyuridine 5'-triphosphate nucleotidohydrolase |
| ORF242 | Vibrio phage VH7D, complete genome | 1923 | 0 | 99% | DNA primase subunit [Vibrio phage nt-1] | 699 | 0 | 93% | DNA primase subunit |
| ORF243 | Vibrio phage VH7D, complete genome | 887 | 0 | 100% | hypothetical protein [Vibrio phage VH7D] | 325 | 3.00E-111 | 100% | – |
| ORF244 | Vibrio phage VH7D, complete genome | 1519 | 0 | 99% | hypothetical protein [Vibrio phage VH7D] | 555 | 0 | 100% | ATP-dependant DNA helicase UvsW |
| ORF245 | Vibrio phage VH7D, complete genome | 1541 | 0 | 99% | hypothetical protein [Vibrio phage VH7D] | 573 | 0 | 100% | – |
| ORF246 | Vibrio phage VH7D, complete genome | 948 | 0 | 99% | putative serine/threonine protein phosphatase [Vibriophage phi-pp2] | 352 | 1.00E-121 | 96% | Putative serine/threonine protein phosphatase |
| ORF247 | Vibrio phage VH7D, complete genome | 826 | 0 | 98% | anaerobic ribonucleoside-triphosphate reductase-activating protein [Vibrio phage VH7D] | 327 | 3.00E-112 | 99% | Anaerobic ribonucleoside-triphosphate reductase-activating protein |
| ORF248 | Vibrio phage VH7D, complete genome | 1099 | 0 | 99% | hypothetical protein [Vibrio phage VH7D] | 407 | 1.00E-142 | 99% | – |
| ORF249 | Vibrio phage VH7D, complete genome | 732 | 0 | 98% | hypothetical protein [Vibrio phage VH7D] | 281 | 1.00E-94 | 97% | – |
| ORF250 | Vibrio phage VH7D, complete genome | 3282 | 0 | 99% | anaerobic ribonucleoside-triphosphate reductase [Vibrio phage VH7D] | 1277 | 0 | 99% | Anaerobic ribonucleoside-triphosphate reductase |
| ORF251 | Vibrio phage VH7D, complete genome | 455 | 1.00E-124 | 100% | hypothetical protein KVP40.0011 [Vibrio phage KVP40] | 142 | 6.00E-42 | 84% | – |
| ORF252 | Vibrio phage VH7D, complete genome | 2577 | 0 | 99% | DNA primase-helicase subunit [Vibrio phage KVP40] | 947 | 0 | 96% | DNA primase-helicase subunit |
| ORF253 | Vibrio phage VH7D, complete genome | 560 | 4.00E-156 | 100% | hypothetical protein [Vibrio phage VH7D] | 204 | 2.00E-65 | 100% | Head assembly gene product |
| ORF254 | Vibrio phage VH7D, complete genome | 2017 | 0 | 99% | recombination and repair protein [Vibrio phage VH7D] | 760 | 0 | 100% | Recombination and repair protein |
| ORF255 | Vibrio phage VH7D, complete genome | 1280 | 0 | 99% | ATP-dependent Clp protease proteolytic subunit 2 [Vibrio phage VH7D] | 496 | 4.00E-176 | 99% | ATP-dependent Clp protease proteolytic subunit 2 |
| ORF256 | Vibrio phage VH7D, complete genome | 992 | 0 | 99% | dihydrofolate reductase [Vibrio phage VH7D] | 374 | 4.00E-130 | 99% | Dihydrofolate reductase |
| ORF257 | Vibrio phage VH7D, complete genome | 1701 | 0 | 99% | single-stranded DNA-binding protein [Vibrio phage VH7D] | 627 | 0 | 99% | Single-stranded DNA-binding protein |
| ORF258 | Vibrio phage VH7D, complete genome | 1037 | 0 | 100% | loader of DNA helicase [Vibrio phage nt-1] | 360 | 1.00E-123 | 92% | Loader of DNA helicase |
| ORF259 | Vibrio phage VH7D, complete genome | 544 | 4.00E-151 | 100% | late promoter transcription accessory protein [Vibrio phage KVP40] | 193 | 3.00E-61 | 95% | Late promoter transcription accessory protein |
| ORF260 | Vibrio phage VH7D, complete genome | 521 | 2.00E-144 | 100% | double-stranded DNA-binding protein [Vibrio phage VH7D] | 186 | 1.00E-58 | 100% | Double-stranded DNA-binding protein |

|  | BLASTn |  |  |  | PSI-BLAST |  |  |  | Description |
| --- | --- | --- | --- | --- | --- | --- | --- | --- | --- |
| ORF Name | Homology | Score | E-Value | Identity (%) | Homology | Score | E-Value | Identity (%) | (With Additional information from Pfam, ScanProsite, NCBI CDD, Interpro) |
| ORF261 | Vibrio phage VH7D, complete genome | 1707 | 0 | 99% | ribonuclease H [Vibrio phage VH7D] | 644 | 0 | 100% | Ribonuclease H |
| ORF262 | Vibrio phage VH7D, complete genome | 5904 | 0 | 98% | long tail fiber proximal subunit [Vibriophage phi-pp2] | 2005 | 0 | 78% | Long tail fiber proximal subunit |
| ORF263 | Vibrio phage VH7D, complete genome | 4233 | 0 | 95% | hypothetical protein pp2_382 [Vibriophage phi-pp2] | 1333 | 0 | 71% | – |
| ORF264 | Vibrio phage VH7D, complete genome | 765 | 0 | 100% | UvsY recombination, repair and single-stranded DNA binding protein [Aeromonas phage Aeh1] | 92.4 | 7.00E-21 | 33% | UvsY recombination, repair and single-stranded DNA binding protein |
| ORF265 | Vibrio phage VH7D, complete genome | 760 | 0 | 99% | hypothetical protein pp2_380 [Vibriophage phi-pp2] | 266 | 7.00E-89 | 96% | – |
| ORF266 | Vibrio phage VH7D, complete genome | 294 | 2.00E-76 | 99% | hypothetical protein KVP40.0380 [Vibrio phage KVP40] | 61.6 | 5.00E-11 | 66% | – |
| ORF267 | Vibrio phage VH7D, complete genome | 316 | 5.00E-83 | 100% | conserved hypothetical protein [Vibrio phage KVP40] | 108 | 2.00E-29 | 93% | – |
| ORF268 | Vibrio phage VH7D, complete genome | 206 | 6.00E-50 | 100% | hypothetical protein [Vibrio phage nt-1] | 58.9 | 2.00E-10 | 78% | – |
| ORF269 | Vibrio phage VH7D, complete genome | 2782 | 0 | 99% | ATP-dependent DNA helicase [Vibrio phage VH7D] | 1060 | 0 | 99% | ATP-dependent DNA helicase |
| ORF270 | Vibrio phage VH7D, complete genome | 388 | 1.00E-104 | 100% | hypothetical protein KVP40.0377 [Vibrio phage KVP40] | 92.4 | 1.00E-22 | 65% | – |
| ORF271 | Vibrio phage VH7D, complete genome | 937 | 0 | 99% | hypothetical protein KVP40.0376 [Vibrio phage KVP40] | 219 | 2.00E-69 | 66% | – |
| ORF272 | Vibrio phage VH7D, complete genome | 904 | 0 | 100% | hypothetical protein KVP40.0375 [Vibrio phage KVP40] | 293 | 1.00E-98 | 85% | – |
| ORF273 | Vibrio phage VH7D, complete genome | 488 | 2.00E-134 | 99% | hypothetical protein pp2_373 [Vibriophage phi-pp2] | 121 | 2.00E-33 | 67% | – |
| ORF274 | Vibrio phage VH7D, complete genome | 1147 | 0 | 99% | hypothetical protein pp2_372 [Vibriophage phi-pp2] | 278 | 2.00E-91 | 63% | – |
| ORF275 | Vibrio phage VH7D, complete genome | 1098 | 0 | 100% | hypothetical protein KVP40.0372 [Vibrio phage KVP40] | 366 | 3.00E-126 | 85% | – |
| ORF276 | Vibrio phage VH7D, complete genome | 1679 | 0 | 99% | hypothetical protein VPFG_00035 [Vibrio phage nt-1] | 548 | 0 | 85% | – |
| ORF277 | Vibrio phage VH7D, complete genome | 2189 | 0 | 100% | hypothetical protein pp2_369 [Vibriophage phi-pp2] | 773 | 0 | 93% | – |
| ORF278 | Vibrio phage VH7D, complete genome | 599 | 9.00E-168 | 99% | hypothetical protein [Vibrio phage VH7D] | 223 | 1.00E-72 | 99% | – |
| ORF279 | Vibrio phage VH7D, complete genome | 898 | 0 | 99% | Inh [Vibriophage phi-pp2] | 222 | 8.00E-71 | 74% | Prohead protease inhibitor |
| ORF280 | Vibrio phage VH7D, complete genome | 1757 | 0 | 96% | tRNA nucleotidyltransferase [Vibriophage phi-pp2] | 612 | 0 | 82% | tRNA nucleotidyltransferase |
| ORF281 | Bacteriophage KVP20 gene for major capsid protein, complete cds | 2843 | 0 | 99% | major capsid protein precursor [Vibrio phage KVP20] | 1050 | 0 | 100% | Major capsid protein precursor |
| ORF282 | Vibrio phage VH7D, complete genome | 1557 | 0 | 99% | major prohead-scaffolding core protein [Vibrio phage VH7D] | 566 | 0 | 100% | Major prohead-scaffolding core protein |
| ORF283 | Vibrio phage VH7D, complete genome | 1181 | 0 | 99% | prohead core protein protease [Vibrio phage VH7D] | 434 | 2.00E-152 | 100% | Prohead core protein protease |
| ORF284 | Vibrio phage VH7D, complete genome | 909 | 0 | 100% | prohead core protein protease [Vibrio phage VH7D] | 317 | 4.00E-108 | 100% | Prohead core protein |
| ORF285 | Vibrio phage VH7D, complete genome | 311 | 2.00E-81 | 100% | hypothetical protein [Vibrio phage VH7D] | 110 | 7.00E-30 | 100% | – |
| ORF286 | Vibrio phage VH7D, complete genome | 2854 | 0 | 99% | structural protein of head [Vibrio phage VH7D] | 1071 | 0 | 100% | T4 like capsid assembly protein |
| ORF287 | Vibrio phage VH7D, complete genome | 926 | 0 | 100% | tail tube protein [Vibrio phage VH7D] | 346 | 2.00E-119 | 100% | T4-like virus tail tube protein |
| ORF288 | Vibrio phage VH7D, complete genome | 3602 | 0 | 99% | tail sheath protein [Vibrio phage VH7D] | 1360 | 0 | 99% | Phage tail sheath protein |
| ORF289 | Vibrio phage VH7D, complete genome | 3325 | 0 | 99% | large terminase protein [Vibrio phage VH7D] | 1213 | 0 | 100% | Large terminase protein |
| ORF290 | Vibrio phage VH7D, complete genome | 1014 | 0 | 100% | terminase DNA packaging enzyme small subunit [Vibrio phage nt-1] | 346 | 8.00E-119 | 93% | Terminase DNA packaging enzyme |
| ORF291 | Vibrio phage VH7D, complete genome | 1003 | 0 | 99% | hypothetical protein [Vibrio phage VH7D] | 363 | 1.00E-125 | 99% | – |
| ORF292 | Vibrio phage VH7D, complete genome | 1724 | 0 | 99% | tail sheath stabilizer and completion protein [Vibrio phage KVP40] | 548 | 6.00E-168 | 77% | Tail sheath stabilizer and completion protein |
| ORF293 | Vibrio phage VH7D, complete genome | 1469 | 0 | 98% | neck protein [Vibrio phage VH7D] | 572 | 0 | 100% | Neck protein |
| ORF294 | Vibrio phage VH7D, complete genome | 1696 | 0 | 99% | neck protein [Vibrio phage VH7D] | 640 | 0 | 100% | Neck protein |
| ORF295 | Vibrio phage VH7D, complete genome | 2760 | 0 | 96% | fibritin [Vibrio phage VH7D] | 1105 | 0 | 98% | Fibritin |
| ORF296 | Vibrio phage VH7D, complete genome | 2582 | 0 | 99% | short tail fiber protein [Vibrio phage VH7D] | 969 | 0 | 100% | Short tail fiber protein |
| ORF297 | Vibrio phage VH7D, complete genome | 2660 | 0 | 98% | hypothetical protein [Vibrio phage VH7D] | 1058 | 0 | 99% | – |
| ORF298 | Vibrio phage VH7D, complete genome | 1242 | 0 | 99% | baseplate wedge subunit and tail pin [Vibrio phage nt-1] | 400 | 5.00E-139 | 84% | Baseplate wedge subunit and tail pin |
| ORF299 | Vibrio phage VH7D, complete genome | 4050 | 0 | 99% | baseplate structural protein [Vibrio phage VH7D] | 1528 | 0 | 100% | Baseplate structural protein |
| ORF300 | Vibrio phage VH7D, complete genome | 1668 | 0 | 98% | baseplate structural protein [Vibrio phage VH7D] | 642 | 0 | 99% | Baseplate structural protein |
| ORF301 | Vibrio phage VH7D, complete genome | 1868 | 0 | 99% | baseplate structural protein [Vibrio phage VH7D] | 699 | 0 | 99% | Baseplate structural protein |
| ORF302 | Vibrio phage VH7D, complete genome | 6250 | 0 | 99% | phage baseplate wedge initiator [Vibriophage phi-pp2] | 2189 | 0 | 90% | Phage baseplate wedge initiator |
| ORF303 | Vibrio phage VH7D, complete genome | 3535 | 0 | 99% | baseplate wedge subunit [Aeromonas phage 44RR2.8t] | 583 | 0 | 46% | Baseplate wedge subunit |
| ORF304 | Vibrio phage VH7D, complete genome | 754 | 0 | 99% | tail lysozyme [Vibrio phage VH7D] | 283 | 1.00E-95 | 100% | Tail lysozyme |
| ORF305 | Vibrio phage VH7D, complete genome | 1546 | 0 | 99% | hypothetical protein KVP40.0339 [Vibrio phage KVP40] | 517 | 0 | 86% | – |
| ORF306 | Vibrio phage VH7D, complete genome | 665 | 0 | 100% | hypothetical protein KVP40.0338 [Vibrio phage KVP40] | 160 | 9.00E-48 | 69% | – |
| ORF307 | Vibrio phage VH7D, complete genome | 532 | 8.00E-148 | 99% | conserved hypothetical protein [Vibrio phage KVP40] | 192 | 7.00E-61 | 96% | – |
| ORF308 | Vibrio phage VH7D, complete genome | 893 | 0 | 99% | hypothetical protein [Vibrio phage VH7D] | 332 | 5.00E-114 | 99% | – |
| ORF309 | Vibrio phage VH7D, complete genome | 2178 | 0 | 99% | tail-associated lysozyme [Vibrio phage VH7D] | 824 | 0 | 99% | Tail-associated lysozyme |
| ORF310 | Vibrio phage VH7D, complete genome | 2239 | 0 | 98% | hypothetical protein [Vibrio phage VH7D] | 863 | 0 | 99% | – |
| ORF311 | Vibrio phage VH7D, complete genome | 1042 | 0 | 99% | baseplate structural protein [Vibrio phage VH7D] | 396 | 2.00E-138 | 100% | Baseplate structural protein |
| ORF312 | Vibrio phage VH7D, complete genome | 1929 | 0 | 97% | phage baseplate tail tube cap [Vibriophage phi-pp2] | 580 | 0 | 78% | Phage baseplate tail tube cap |
| ORF313 | Vibrio phage VH7D, complete genome | 804 | 0 | 98% | head completion protein [Salmonella phage S16] | 202 | 2.00E-63 | 64% | Head completion protein |
| ORF314 | Vibrio phage VH7D, complete genome | 1081 | 0 | 99% | DNA end protector protein [Vibrio phage nt-1] | 363 | 6.00E-125 | 91% | DNA end protector protein |
| ORF315 | Vibrio phage VH7D, complete genome | 1347 | 0 | 99% | phage baseplate-tail tube initiator [Vibriophage phi-pp2] | 455 | 1.00E-159 | 87% | Phage baseplate-tail tube initiator |
| ORF316 | Vibrio phage VH7D, complete genome | 1463 | 0 | 98% | gp26 baseplate hub subunit [Aeromonas phage Aeh1] | 117 | 2.00E-27 | 28% | Baseplate hub subunit |
| ORF317 | Vibrio phage VH7D, complete genome | 311 | 2.00E-81 | 100% | hypothetical protein VPFG_00381 [Vibrio phage nt-1] | 108 | 3.00E-29 | 95% | – |
| ORF318 | Vibrio phage VH7D, complete genome | 3530 | 0 | 99% | hypothetical protein KVP40.0326 [Vibrio phage KVP40] | 1147 | 0 | 86% | – |
| ORF319 | Vibrio phage VH7D, complete genome | 926 | 0 | 98% | tail completion and sheath stabilizer protein [Vibrio phage KVP40] | 363 | 1.00E-125 | 97% | Tail completion and sheath stabilizer protein |
| ORF320 | Vibrio phage VH7D, complete genome | 1181 | 0 | 99% | dNMP kinase [Vibrio phage KVP40] | 375 | 2.00E-129 | 85% | dNMP kinase |
| ORF321 | Vibrio phage VH7D, complete genome | 494 | 3.00E-136 | 100% | hypothetical protein KVP40.0323 [Vibrio phage KVP40] | 156 | 4.00E-47 | 89% | – |
| ORF322 | Vibrio phage VH7D, complete genome | 837 | 0 | 99% | hypothetical protein VPFG_00376 [Vibrio phage nt-1] | 280 | 7.00E-94 | 87% | – |
| ORF323 | Vibrio phage VH7D, complete genome | 466 | 7.00E-128 | 99% | hypothetical protein KVP40.0321 [Vibrio phage KVP40] | 161 | 2.00E-49 | 96% | – |
| ORF324 | Vibrio phage VH7D, complete genome | 649 | 0 | 98% | hypothetical protein pp2_320 [Vibriophage phi-pp2] | 236 | 2.00E-77 | 92% | – |
| ORF325 | Vibrio phage VH7D, complete genome | 405 | 1.00E-109 | 98% | hypothetical protein KVP40.0318 [Vibrio phage KVP40] | 131 | 9.00E-38 | 84% | – |

BLASTn PSI-BLAST Description

ORF Name Homology Score E-Value Identity (%) Homology Score E-Value Identity (%) (With Additional information from Pfam, ScanProsite, NCBI CDD, Interpro)

| ORF326 | Vibrio phage VH7D, complete genome | 909 | 0 | 99% | hypothetical protein KVP40.0317 [Vibrio phage KVP40] | 277 | 7.00E-92 | 74% | – |
| --- | --- | --- | --- | --- | --- | --- | --- | --- | --- |
| ORF327 | No Significant Homology | – | – | – | No Significant Homology | – | – | – | – |
| ORF328 | Vibrio phage VH7D, complete genome | 405 | 1.00E-109 | 97% | hypothetical protein pp2_317 [Vibriophage phi-pp2] | 144 | 2.00E-42 | 93% | – |
| ORF329 | No Significant Homology | – | – | – | No Significant Homology | – | – | – | – |
| ORF330 | No Significant Homology | – | – | – | No Significant Homology | – | – | – | – |
| ORF331 | Vibrio phage VH7D, complete genome | 287 | 3.00E-74 | 99% | hypothetical protein pp2_312 [Vibriophage phi-pp2] | 75.5 | 3.00E-16 | 80% | – |
| ORF332 | Vibriophage phi-pp2, complete genome | 257 | 4.00E-65 | 86% | hypothetical protein KVP40.0312 [Vibrio phage KVP40] | 145 | 3.00E-43 | 88% | – |
| ORF333 | No Significant Homology | – | – | – | hypothetical protein [Vibrio phage VH7D] | 994 | 0 | 83% | – |
| ORF334 | No Significant Homology | – | – | – | hypothetical protein [Vibrio phage VH7D] | 216 | 7.00E-70 | 88% | – |
| ORF335 | No Significant Homology | – | – | – | hypothetical protein KVP40.0310 [Vibrio phage KVP40] | 132 | 2.00E-36 | 50% | – |
| ORF336 | Vibrio phage VH7D, complete genome | 289 | 1.00E-74 | 92% | hypothetical protein pp2_307 [Vibriophage phi-pp2] | 62.8 | 4.00E-11 | 87% | – |
| ORF337 | Vibrio phage VH7D, complete genome | 316 | 7.00E-83 | 91% | hypothetical protein pp2_306 [Vibriophage phi-pp2] | 89.7 | 2.00E-21 | 65% | – |
| ORF338 | Vibrio phage VH7D, complete genome | 394 | 3.00E-106 | 100% | hypothetical protein [Vibrio phage VH7D] | 141 | 7.00E-42 | 100% | – |
| ORF339 | Vibrio phage VH7D, complete genome | 682 | 0 | 99% | hypothetical protein [Vibrio phage VH7D] | 247 | 9.00E-82 | 99% | – |
| ORF340 | Vibrio phage VH7D, complete genome | 638 | 2.00E-179 | 99% | hypothetical protein VPFG_00355 [Vibrio phage nt-1] | 189 | 5.00E-59 | 76% | – |
| ORF341 | Vibrio phage VH7D, complete genome | 671 | 0 | 99% | conserved hypothetical protein [Vibrio phage KVP40] | 236 | 2.00E-77 | 93% | – |
| ORF342 | Vibrio phage VH7D, complete genome | 621 | 2.00E-174 | 100% | hypothetical protein pp2_301 [Vibriophage phi-pp2] | 213 | 6.00E-69 | 89% | – |
| ORF343 | Vibrio phage VH7D, complete genome | 582 | 8.00E-163 | 100% | hypothetical protein pp2_300 [Vibriophage phi-pp2] | 135 | 2.00E-38 | 59% | – |
| ORF344 | Vibrio phage VH7D, complete genome | 2388 | 0 | 97% | hypothetical protein KVP40.0300 [Vibrio phage KVP40] | 966 | 0 | 96% | Domain of unknown function (DUF2828) |
| ORF345 | Vibrio phage VH7D, complete genome | 926 | 0 | 98% | conserved hypothetical protein [Vibrio phage KVP40] | 347 | 2.00E-119 | 94% | Protein of unknown function (DUF458) |
| ORF346 | Vibrio phage VH7D, complete genome | 2820 | 0 | 99% | tail fiber fragment [Vibriophage phi-pp2] | 137 | 5.00E-31 | 88% | Tail fiber fragment |
| ORF347 | Vibrio phage VH7D, complete genome | 5009 | 0 | 93% | phage minor structural protein [Bacillus thuringiensis] | 56.2 | 2.00E-04 | 29% | Phage minor structural protein |
| ORF348 | Vibrio phage VH7D, complete genome | 2327 | 0 | 91% | long tail fiber distal subunit [Vibrio phage KVP40] | 782 | 9.00E-68 | 40% | Long tail fiber distal subunit |
| ORF349 | Vibrio phage VH7D, complete genome | 6517 | 0 | 94% | long tail fiber distal subunit [Vibrio phage nt-1] | 1039 | 0 | 58% | Long tail fiber distal subunit |
| ORF350 | Vibrio phage VH7D, complete genome | 1003 | 0 | 98% | hypothetical protein pp2_295 [Vibriophage phi-pp2] | 337 | 4.00E-115 | 87% | – |
| ORF351 | Vibrio phage VH7D, complete genome | 521 | 2.00E-144 | 98% | hypothetical protein [Vibrio phage VH7D] | 205 | 6.00E-66 | 99% | Protein of unknown function (DUF2829) |
| ORF352 | Vibrio phage VH7D, complete genome | 538 | 2.00E-149 | 99% | anti-sigma70 protein [Vibrio phage KVP40] | 196 | 1.00E-62 | 93% | Anti-sigma70 protein |
| ORF353 | Vibrio phage VH7D, complete genome | 1330 | 0 | 99% | hypothetical protein [Vibrio phage VH7D] | 505 | 9.00E-180 | 99% | – |
| ORF354 | Vibrio phage VH7D, complete genome | 366 | 5.00E-98 | 99% | No Significant Homology | – | – | – | – |
| ORF355 | Vibrio phage VH7D, complete genome | 555 | 2.00E-154 | 99% | hypothetical protein [Vibrio phage VH7D] | 208 | 3.00E-67 | 100% | – |
| ORF356 | Vibrio phage VH7D, complete genome | 1286 | 0 | 99% | PhoH-like protein [Vibrio phage VH7D] | 484 | 9.00E-172 | 100% | PhoH-like protein |
| ORF357 | Vibrio phage VH7D, complete genome | 854 | 0 | 98% | hypothetical protein [Vibrio phage VH7D] | 338 | 1.00E-116 | 99% | Peptidase Zinc Binding Domain |
| ORF358 | Vibrio phage VH7D, complete genome | 1857 | 0 | 97% | hypothetical protein [Vibrio phage VH7D] | 738 | 0 | 98% | Poly(ADP-ribose) polymerase |
| ORF359 | Vibrio phage VH7D, complete genome | 483 | 7.00E-133 | 99% | hypothetical protein [Vibrio phage VH7D] | 176 | 1.00E-54 | 100% | – |
| ORF360 | Vibrio phage VH7D, complete genome | 499 | 7.00E-138 | 100% | hypothetical protein [Vibrio phage VH7D] | 183 | 1.00E-57 | 100% | – |
| ORF361 | Vibrio phage VH7D, complete genome | 549 | 8.00E-153 | 99% | hypothetical protein [Vibrio phage VH7D] | 187 | 3.00E-59 | 99% | – |
| ORF362 | Vibrio phage VH7D, complete genome | 289 | 9.00E-75 | 100% | hypothetical protein [Vibrio phage VH7D] | 102 | 7.00E-27 | 100% | – |
| ORF363 | Vibrio phage VH7D, complete genome | 451 | 2.00E-123 | 98% | hypothetical protein [Vibrio phage VH7D] | 166 | 5.00E-51 | 98% | – |
| ORF364 | Vibrio phage VH7D, complete genome | 1591 | 0 | 99% | radical SAM domain-containing protein [Vibrio phage nt-1] | 506 | 3.00E-178 | 82% | Radical SAM domain-containing protein |
| ORF365 | Vibrio phage VH7D, complete genome | 444 | 3.00E-121 | 99% | hypothetical protein [Vibrio phage VH7D] | 170 | 9.00E-53 | 100% | – |
| ORF366 | Vibrio phage VH7D, complete genome | 2333 | 0 | 99% | DNA topoisomerase medium subunit [Vibrio phage VH7D] | 878 | 0 | 99% | DNA topoisomerase medium subunit |
| ORF367 | Vibrio phage VH7D, complete genome | 1812 | 0 | 99% | thioredoxin [Vibriophage phi-pp2] | 620 | 0 | 92% | Thioredoxin |
| ORF368 | Vibrio phage VH7D, complete genome | 339 | 1.00E-89 | 99% | hypothetical protein [Vibrio phage VH7D] | 130 | 1.00E-37 | 100% | – |
| ORF369 | Vibrio phage VH7D, complete genome | 1031 | 0 | 99% | transglycosylase SLT domain protein [Vibrio phage KVP40] | 358 | 4.00E-123 | 89% | Transglycosylase SLT domain protein |
| ORF370 | Vibrio phage VH7D, complete genome | 549 | 8.00E-153 | 99% | thioredoxin [Vibrio phage KVP40] | 192 | 4.00E-61 | 91% | Thioredoxin |
| ORF371 | Vibrio phage VH7D, complete genome | 2061 | 0 | 99% | ribonucleoside-diphosphate reductase 1 subunit beta [Vibrio phage VH7D] | 783 | 0 | 100% | Nibonucleoside-diphosphate reductase 1 subunit beta |
| ORF372 | Vibrio phage VH7D, complete genome | 4067 | 0 | 99% | ribonucleoside-diphosphate reductase subunit alpha [Vibrio phage VH7D] | 1547 | 0 | 99% | Nibonucleoside-diphosphate reductase subunit alpha |
| ORF373 | Vibrio phage VH7D, complete genome | 859 | 0 | 99% | hypothetical protein [Vibrio phage VH7D] | 325 | 2.00E-111 | 99% | – |
| ORF374 | Vibrio phage VH7D, complete genome | 377 | 3.00E-101 | 100% | hypothetical protein [Vibrio phage VH7D] | 134 | 4.00E-39 | 100% | – |
| ORF375 | Vibrio phage VH7D, complete genome | 427 | 3.00E-116 | 100% | hypothetical protein pp2_269 [Vibriophage phi-pp2] | 113 | 2.00E-30 | 78% | – |
| ORF376 | Vibrio phage VH7D, complete genome | 926 | 0 | 99% | hypothetical protein [Vibrio phage VH7D] | 340 | 4.00E-117 | 99% | – |
| ORF377 | Vibrio phage VH7D, complete genome | 1308 | 0 | 99% | hypothetical protein [Vibrio phage VH7D] | 486 | 2.00E-172 | 100% | Prokaryotic membrane lipoprotein lipid attachment site |
| ORF378 | Vibrio phage VH7D, complete genome | 472 | 2.00E-129 | 99% | hypothetical protein [Vibrio phage VH7D] | 183 | 1.00E-57 | 99% | – |
| ORF379 | Vibrio phage VH7D, complete genome | 2189 | 0 | 99% | hypothetical protein [Vibrio phage VH7D] | 845 | 0 | 99% | – |
| ORF380 | Vibrio phage VH7D, complete genome | 604 | 2.00E-169 | 100% | hypothetical protein [Vibrio phage VH7D] | 222 | 2.00E-72 | 100% | – |
| ORF381 | Vibrio phage VH7D, complete genome | 2338 | 0 | 95% | nicotinamide phosphoribosyl transferase [Vibrio phage VH7D] | 1009 | 0 | 98% | Nicotinamide phosphoribosyl transferase |
| ORF382 | Vibrio phage VH7D, complete genome | 898 | 0 | 99% | hypothetical protein [Vibrio phage VH7D] | 345 | 7.00E-119 | 99% | – |
| ORF383 | Vibrio phage VH7D, complete genome | 837 | 0 | 95% | hypothetical protein [Vibrio phage VH7D] | 346 | 4.00E-119 | 97% | – |
| ORF384 | Vibrio phage VH7D, complete genome | 1380 | 0 | 99% | hypothetical protein [Vibrio phage VH7D] | 532 | 0 | 99% | – |
| ORF385 | Vibrio phage VH7D, complete genome | 1214 | 0 | 99% | hypothetical protein [Vibrio phage VH7D] | 471 | 1.00E-166 | 100% | – |
| ORF386 | Vibrio phage VH7D, complete genome | 654 | 0 | 94% | hypothetical protein [Vibrio phage VH7D] | 281 | 1.00E-94 | 95% | – |
| ORF387 | Vibrio phage VH7D, complete genome | 311 | 3.00E-81 | 89% | hypothetical protein [Vibrio phage VH7D] | 158 | 5.00E-48 | 91% | – |
| ORF388 | Vibrio phage VH7D, complete genome | 610 | 4.00E-171 | 100% | hypothetical protein [Vibrio phage VH7D] | 225 | 1.00E-73 | 100% | Protein of unknown function (DUF3307) |
| ORF389 | Vibrio phage VH7D, complete genome | 676 | 0 | 93% | hypothetical protein [Vibrio phage VH7D] | 311 | 7.00E-106 | 96% | – |
| ORF390 | Vibriophage phi-pp2, complete genome | 464 | 5.00E-127 | 85% | hypothetical protein pp2_242 [Vibriophage phi-pp2] | 281 | 3.00E-94 | 84% | – |
